# Diffusion, Crowding & Protein Stability in a Dynamic Molecular Model of the Bacterial Cytoplasm

**DOI:** 10.1371/journal.pcbi.1000694

**Published:** 2010-03-05

**Authors:** Sean R. McGuffee, Adrian H. Elcock

**Affiliations:** Department of Biochemistry, University of Iowa, Iowa City, Iowa, United States of America; University of Houston, United States of America

## Abstract

A longstanding question in molecular biology is the extent to which the behavior of macromolecules observed *in vitro* accurately reflects their behavior *in vivo*. A number of sophisticated experimental techniques now allow the behavior of individual types of macromolecule to be studied directly *in vivo*; none, however, allow a wide range of molecule types to be observed simultaneously. In order to tackle this issue we have adopted a computational perspective, and, having selected the model prokaryote *Escherichia coli* as a test system, have assembled an atomically detailed model of its cytoplasmic environment that includes 50 of the most abundant types of macromolecules at experimentally measured concentrations. Brownian dynamics (BD) simulations of the cytoplasm model have been calibrated to reproduce the translational diffusion coefficients of Green Fluorescent Protein (GFP) observed *in vivo*, and “snapshots” of the simulation trajectories have been used to compute the cytoplasm's effects on the thermodynamics of protein folding, association and aggregation events. The simulation model successfully describes the relative thermodynamic stabilities of proteins measured in *E. coli*, and shows that effects additional to the commonly cited “crowding” effect must be included in attempts to understand macromolecular behavior *in vivo*.

## Author Summary

The interior of a typical bacterial cell is a highly crowded place in which molecules must jostle and compete with each other in order to carry out their biological functions. The conditions under which such molecules are typically studied *in vitro*, however, are usually quite different: one or a few different types of molecules are studied as they freely diffuse in a dilute, aqueous solution. There is therefore a significant disconnect between the conditions under which molecules can be most usefully studied and the conditions under which such molecules usually “live”, and developing ways to bridge this gap is likely to be important for properly understanding molecular behavior *in vivo*. Toward this end, we show in this work that computer simulations can be used to model the interior of bacterial cells at a near atomic level of detail: the rates of diffusion of proteins are matched to known experimental values, and their thermodynamic stabilities are found to be in good agreement with the few measurements that have so far been performed *in vivo*. While the simulation approach is certainly not free of assumptions, it offers a potentially important complement to experimental techniques and provides a vivid illustration of molecular behavior inside a biological cell that is likely to be of significant educational value.

## Introduction

While reductionist biophysical studies continue to contribute important insights into the properties and functions of biological macromolecules, research attention is increasingly being directed at uncovering the extent to which behavior observed *in vitro* is likely to reflect that occurring *in vivo* [1],[2]. In a physiological setting, all biomolecules must inevitably experience non-specific, unintended interactions with the intracellular milieu and there are good theoretical reasons to expect that, even if such interactions are only steric in nature, significant alterations in macromolecular folding and association equilibria may result [2],[3]. In order to allow macromolecules to be directly interrogated *in vivo* therefore, a number of important developments have been made in the experimental fields of hydrogen exchange [4], nuclear magnetic resonance [5],[6], and fluorescence spectroscopies [7]–[9].

An alternative to the use of experimental techniques is to assemble a molecular model of an intracellular environment *in silico* and to use molecular simulation techniques to explore its behavior; if such a model could be shown to be realistic – and that is a big ‘if’ – it would have the important advantage of allowing the simultaneous, direct observation of *all* molecules in the system. In fact, at least two simulation studies that attempt to model the bacterial cytoplasm have already been reported [10],[11], producing a number of intriguing results. Both of these previous studies, however, modeled all cytoplasmic molecules as spheres and it is perhaps to be anticipated therefore that simulations that include structurally detailed macromolecular models might lead to additional insights. In pursuit of this strategy, we have chosen the gram-negative prokaryote *Escherichia coli* as a test system, combining quantitative proteomic [12] and high-resolution structural data [13] to build a first structurally detailed molecular model of the bacterial cytoplasm.

## Results

**Figure 1:**
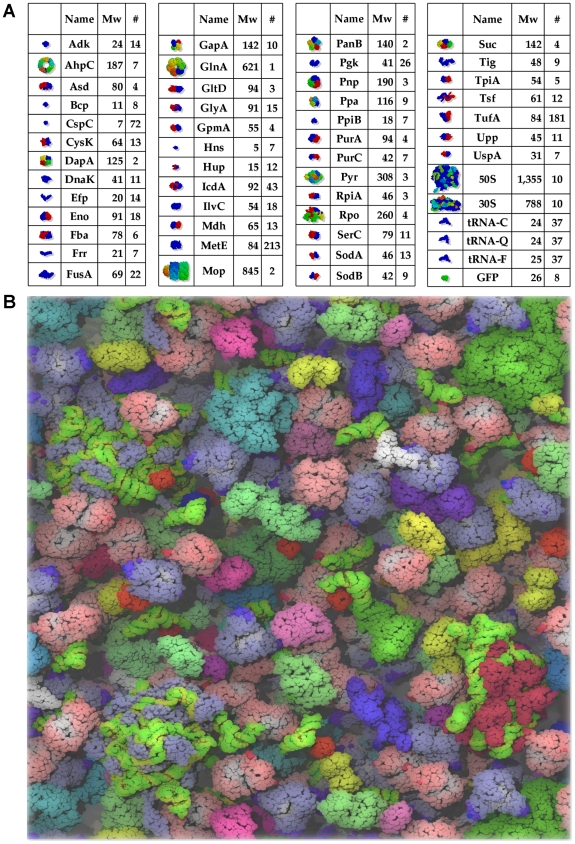
The cytoplasm model. **A**. Schematic inventory of the contents of the cytoplasm model. **B**. Rendering of the cytoplasm model at the end of a Brownian dynamics simulation performed with the ‘full’ energy model (see text). RNA is shown as green and yellow. This figure was prepared with VMD [110].

Full details of the construction of the model are provided in Methods. Briefly, however, it is to be noted that the model contains 50 different types of the most abundant macromolecules of the *E. coli* cytoplasm (accounting for ∼85% of the cytoplasm's *characterized* protein content by weight; [12]) and a total of 1008 individual molecules. Eight of these molecules are copies of the heterologous (non-*E. coli*) protein GFP (Green Fluorescent Protein), which has been included so that the diffusional characteristics of the model can be compared with *in vivo* experimental results (see below). A snapshot of the modeled system, together with a full listing of its constituents, is shown in Figure 1; the total combined macromolecular concentration in all of the simulations reported here is 275g/l.

### Parameterization of the simulation model

Starting from three different randomized initial configurations of the cytoplasm model (all shown in Figure S1), we performed independent Brownian dynamics (BD) simulations [14] to explore diffusive behavior. A variety of energetic descriptions of intermolecular interactions were explored, ranging from a simple steric-only model – which provides an opportunity to directly test the predictions of excluded-volume ‘crowding’ theories [2],[3] – to models that include both long-range electrostatic interactions and short-range potential functions that mimic hydrophobic interactions between exposed non-polar groups. In order to determine the most realistic energy model, the long-time translational diffusion coefficients, D^L^_trans_, of the ‘tracer’ GFP molecules were computed from the BD simulations and compared with previously reported experimental estimates obtained by fluorescence-recovery-after-photobleaching (FRAP) analysis of GFP in the *E. coli* cytoplasm [15]–[18].

**Figure 2:**
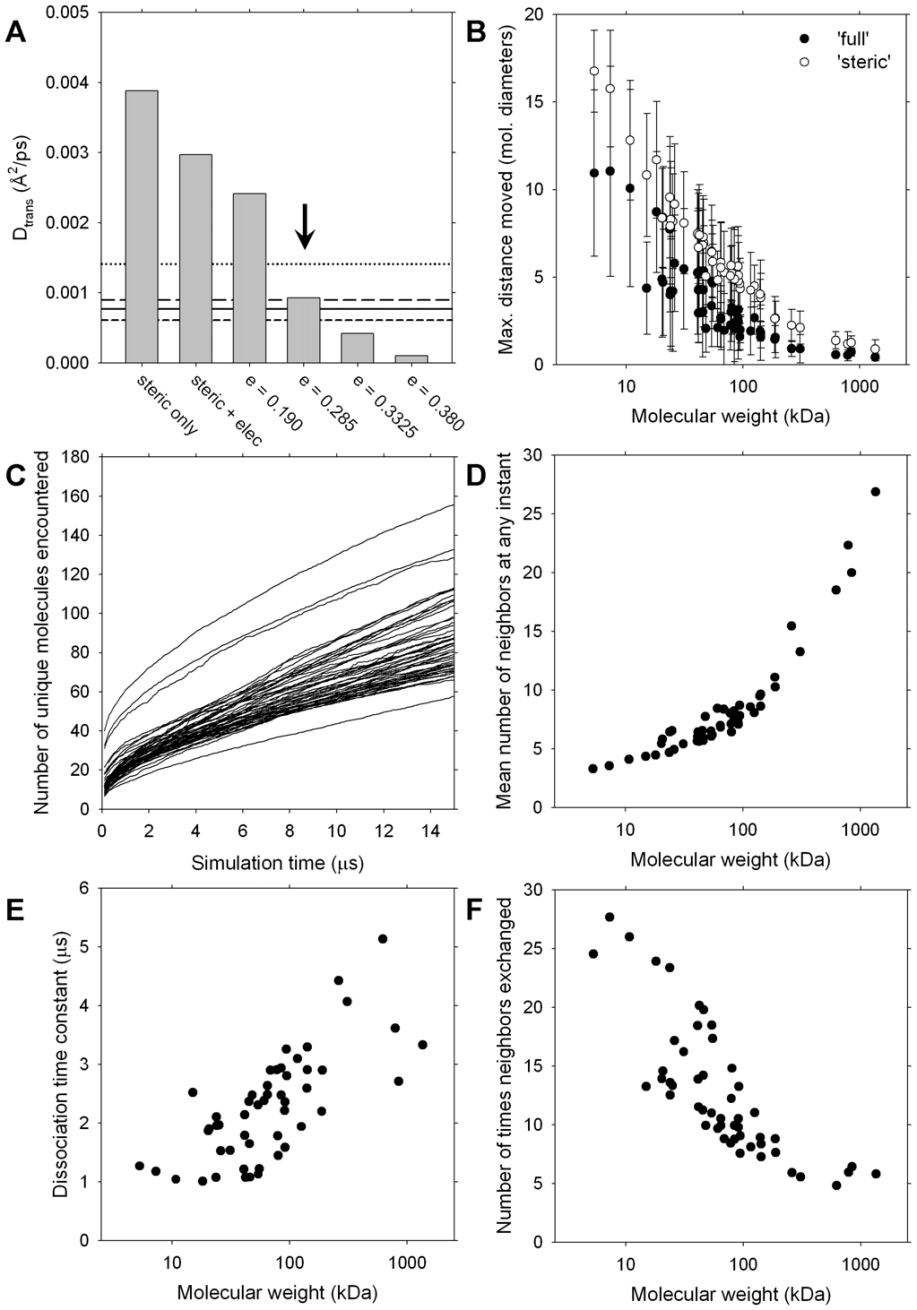
Parameterization and sampling in the cytoplasm model. **A**. Extrapolated long-time D_trans_ values for GFP from BD simulations performed with different energy models; ‘ε’ refers to the well-depth (in kcal/mol) of the Lennard-Jones potential used to describe hydrophobic interactions (see Methods). Solid, long-dash, short-dash and dotted lines are the experimental D_trans_ values from refs. 14, 15, 16 and 17 respectively. The vertical arrow indicates the energy model selected for further BD simulation. **B**. Average of the maximum distance moved during the 15µs of production for all molecule types plotted versus their molecular weights. Upper error bars indicate the largest value of the maximum distance moved found for any molecule of that type; lower error bars indicate the smallest value of the maximum distance moved. All distances expressed in terms of the molecular diameters (obtained by doubling the hydrodynamic radius calculated by HydroPro [88]. **C**. Average number of unique neighbors encountered by each molecule type as a function of simulation time; each line refers to a different molecule type. **D**. Average number of neighbors possessed by each molecule type at any instant, plotted versus molecular weight. **E**. Time constant for the slower of the two exponentials describing the rate at which neighbors are lost, plotted for each molecule type versus molecular weight. **F**. Average number of times that each molecule type's immediate neighbors exchange during 15µs simulation plotted versus molecular weight of each molecule type.

A comparison of the computed GFP D^L^_trans_ values obtained with the different energy models is shown in Figure 2A. For simulations in which only steric interactions operate between macromolecules the computed GFP D^L^_trans_ value is 3–6 times higher than the experimental estimates, and although this value decreases somewhat when electrostatic interactions between macromolecules are added, it remains 2–5 times too high relative to experiment. A more realistic model of macromolecular interactions would allow favorable short-range attractions to occur between exposed hydrophobic atoms and one simple way of approximating such interactions is to use a Lennard-Jones potential, with the well-depth of the potential, ε, being treated as an adjustable parameter (see Methods). As shown in Figure 2A, the inclusion of such a term results in computed GFP D^L^_trans_ values that decrease monotonically as the well-depth, ε, increases in magnitude. The best agreement with experiment is obtained with ε = 0.285 kcal/mol: at this value of ε the computed value of D^L^_trans_ – which is ∼10% of its value at infinite dilution – is within the experimental error of all *in vivo* estimates [15]–[18] including a very recent report for diffusion in cells growing in minimal media [18]. As noted in the Discussion, this optimal value of ε is very similar to the values obtained in our previous efforts to model the interaction thermodynamics of single-component protein solutions [19].

Having determined that good agreement with experiment could be obtained using a so-called ‘full’ energy model that included steric, electrostatic and short-range attractive hydrophobic interactions, we extended each of three independent simulations performed with this energy model to 20µs (see Figure S2 for plots of the system's energy versus time). In order to provide a useful baseline for comparative purposes we also performed extended simulations with the purely ‘steric’ energy model (i.e. one that neglects the electrostatic and hydrophobic interactions); the latter simulations were performed for simulation times of 17.5µs. Each BD simulation using the ‘full’ energy model required more than a year (clock-time) to complete. For both energy models, snapshots taken from the last 15µs of each simulation were used for detailed analysis.

### Overall characteristics of the Brownian dynamics simulations

An informative, albeit non-quantitative, impression of the simulation behavior can be obtained by viewing movies of the simulations (Supporting Information). In some respects, these movies can be considered a key result of this work: they represent, in effect, dynamic analogs of the highly influential pictorial representations pioneered by Goodsell [20]. Examination of a typical movie obtained from a simulation performed with the ‘steric’ energy model shows the simulated motion to be rapid, chaotic and obviously Brownian. For the more realistic ‘full’ model, on the other hand, motion is somewhat slower-paced, and molecules can be seen to fluctuate between engagement in short-lived associations and periods of relatively free diffusion.

We can place these observations on a more quantitative footing, and obtain an indication of the extent of sampling achieved in 15µs of simulation, from the remaining panels of Figure 2. Figure 2B shows the maximum distances moved, on average, by each molecule type during simulations performed with the ‘full’ and ‘steric’ energy models; all distances are expressed relative to the diameter of the diffusing molecule. In the case of GFP with the ‘full’ energy model, for example, each molecule travels, on average, approximately 6 molecular diameters (i.e. 320Å) from its position at the beginning of the simulation. Since the data in Figure 2B are plotted versus molecular weight it is apparent that 15µs of simulation is sufficient for the smaller macromolecules to move very significant distances, while for the largest macromolecules (the 30S and 50S ribosomal subunits), little motion away from the initial position is achieved. On this basis alone, therefore, we expect the estimates of diffusional behavior for the smaller macromolecules to be somewhat more precise than those of the larger macromolecules. A second measure of the extent of sampling achieved during each simulation period is provided by plotting the number of unique interaction partners encountered by each type of macromolecule as a function of the simulation time (Figure 2C). Encouragingly, most molecule types encounter many unique neighbors over the course of 15µs: during a typical simulation with the ‘full’ model, for example, each GFP molecule encounters ∼80 different neighbors. Just as importantly, the total numbers of unique neighbors continues to increase even toward the end of the simulation period: this indicates that the cytoplasm model remains highly dynamic and does not tend to ‘freeze’ as the simulation progresses.

As might be expected, the average numbers of neighbors that a macromolecule possesses at any instant scales essentially monotonically with its molecular weight: the average number of macromolecules in the immediate neighborhood of a GFP molecule, for example, is only ∼5 while for the 50S ribosomal subunit it is more than 25 (Figure 2D). The time constants for the dissociation of these neighboring interactions – which in all cases are in the microsecond range – also scale straightforwardly with the molecular weight (Figure 2E), indicating that molecules remain in the neighborhood of larger macromolecules for somewhat longer periods of time than they do with smaller macromolecules. The data shown in Figures 2C and 2D can be combined to provide an estimate of the number of times that each molecule's entire complement of neighbors is replaced during the simulations (Figure 2F). Interestingly, while the overall trend is such that smaller macromolecules encounter a more dynamic constellation of neighbors even the largest macromolecules experience a significant number of environmental changes during the 15µs simulation period. While each GFP molecule, for example, effectively ‘shed its skin’ of neighbors a total of ∼14 times, even the 50S ribosomal subunit undergoes ∼5 such transformations (Fig. 2F). This observation suggests that the limited diffusional exploration carried out by the largest macromolecules evident in Figure 2B may, in at least one important respect, give a misleadingly low indication of the extent of configurational sampling achieved in the simulations: it is in fact, possible for a completely static macromolecule to rapidly encounter widely different microenvironments simply by virtue of the dynamic exchange of its smaller, more mobile neighbors.

### Translational and rotational diffusion

**Figure 3:**
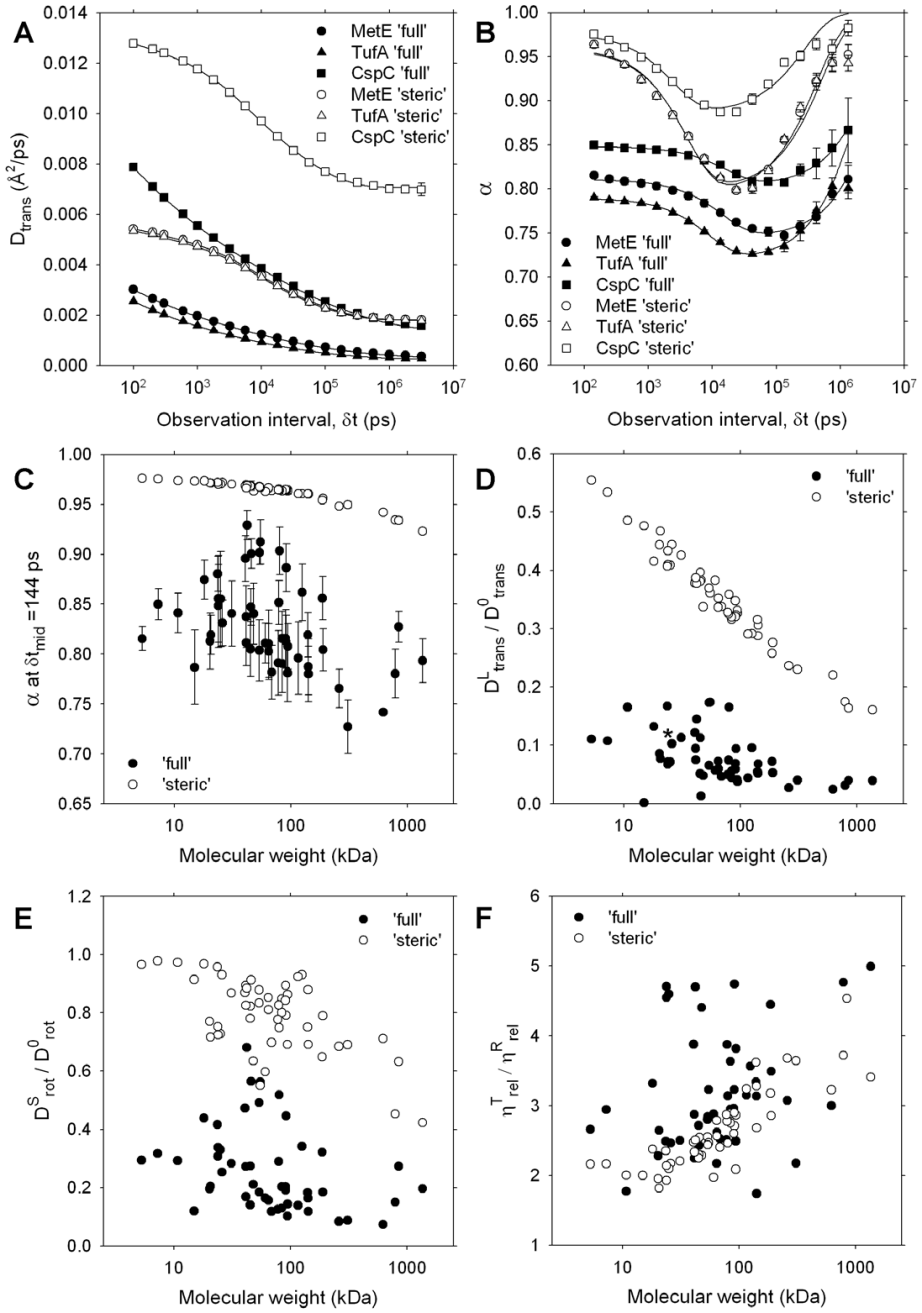
Translational and rotational diffusion in the cytoplasm model. **A**. D_trans_ values for the three most abundant proteins plotted versus observation interval δt; error bars indicate the standard deviation of values obtained from three independent simulations; solid lines represent fits to the data obtained by integrating the analytical functions shown in the next panel. **B**. Computed anomality exponents, α, obtained by numerically differentiating the D_trans_ values shown in **A**; solid lines represent fits to the data using an analytical function defined in Methods. **C**. Anomality exponent, α, computed at the shortest accessible time interval (δt_mid_ = 144ps) plotted for all molecule types versus molecular weight; error bars represent standard deviations from the three independent BD simulations. **D**. Long-time D_trans_ values expressed relative to infinite-dilution values plotted versus molecular weight of each molecule type; asterisk denotes GFP. **E**. Short-time D_rot_ values expressed relative to infinite-dilution values plotted versus molecular weight of each molecule type. **F**. Ratio of the effective translational and effective rotational viscosities, plotted for all molecule types versus molecule weight.

While it was noted above that the long-time D^L^_trans_ value of GFP obtained with the ‘full’ energy model is in good agreement with *in vivo* measurements (Figure 2A), there are other aspects of diffusional behavior in the simulations that warrant examination. One question that is of interest is how the observed D_trans_ values of macromolecules depend on the observation interval, δt, over which their diffusion is monitored (see Methods). The answer to this question is plotted in Figures 3A and 3B for the three most abundant members of the cytoplasm model (MetE, TufA and CspC); these proteins have been chosen for closer examination because their high abundance yields the most statistically robust numbers, but very similar results are obtained for the other constituents of the model. Figure 3A plots the computed D_trans_ values of the three proteins versus δt for both the ‘full’ and ‘steric’ energy models. The clear variation of D_trans_ with δt seen for all three proteins is indicative of ‘anomalous’ diffusion [21]–; the magnitude of the anomaly is conventionally expressed by the anomality exponent, α, (Methods) which is plotted for the same proteins, again versus δt, in Figure 3B. Examination of this figure shows that with the ‘steric’ energy model, the diffusion of all three proteins progresses from being normal (α∼1), to transiently subdiffusive (α<1), to normal again as the observation interval increases from δt∼100ps to δt∼10ns to δt∼1µs. With the ‘full’ model, in contrast, macromolecules exhibit transiently anomalous subdiffusion even at the shortest observation intervals; again however, a slow, but unequivocal return toward normal diffusion occurs on a high microsecond timescale. The same qualitative features are seen for all other molecule types although, for the largest macromolecules or those with the very lowest copy numbers, it is not always clear that sampling is sufficient to be absolutely certain of a return to normal diffusion at the longest δt values. At very short values of δt however we can obtain quite precise values of α for all molecule types; when these are plotted versus molecular weight (Figure 3C) it is apparent that while there is a clear difference between the values obtained with the two energy models, and a clear size-dependence of α with the ‘steric’ model, there is no such obvious trend with the ‘full’ model.

For both energy models, the plots of α versus δt fit well to an analytical function (solid lines in Figure 3B) that, when integrated, enables an asymptotic *long-time* translational diffusion coefficient, D^L^_trans_, to be estimated (see Methods). The observed D^L^_trans_ values of all molecule types are expressed relative to their translational diffusion coefficients at infinite dilution (D^0^_trans_) and plotted versus molecular weight in Figure 3D. For both energy models, the ratio D^L^_trans_/D^0^_trans_ decreases with increasing molecular weight, which is qualitatively consistent with experimental studies of tracer protein diffusion in simple single-component protein solutions [24] and of Ficoll diffusion in the cytoplasm of mouse 3T3 cells [25]. The poorer correlation obtained for the ‘full’ model (which does not appear to be solely due to incomplete sampling) suggests that translational diffusion *in vivo* should not be predictable with arbitrary precision solely from knowledge of molecular weight; again, this is in line with the often significant variations observed in the *in vivo* diffusion coefficients of similarly-sized GFP-constructs [15],[26]. It is perhaps worth noting, however, that the computed diffusive behavior of the heterologous GFP – marked by an asterisk in the ‘full’ model data points – is consistent with the general trend established by the endogenous *E. coli* macromolecules.

The rotational motion of macromolecules is also significantly affected by immersion in the cytoplasm model. In the case of the ‘full’ energy model, the rotational behavior can be fit equally well by either a double-exponential function or a model that describes transiently anomalous rotational diffusion [27]. Since it is the rotational behavior on a nanosecond timescale that is more relevant to experimental measurements (see Methods), we plot the short-time rotational diffusion coefficient, D^S^_rot_ of all molecule types, relative to their rotational diffusion coefficients at infinite dilution, D^0^_rot_, in Figure 3E. As would be anticipated given the translational behavior shown above, rotational diffusion is significantly slower with the ‘full’ model than it is with the ‘steric’ model.

Notably, a comparison of Figures 3D and 3E shows that with both energy models rotational diffusion is slowed *less* by immersion in the cytoplasm than is translational diffusion. This can be viewed as indicating that the two kinds of motion experience different relative viscosities (η_rel_^T^ and η_rel_^R^ for translational and rotational diffusion respectively). Figure 3F plots the ratio of these relative viscosities, η_rel_^T^/η_rel_^R^, versus molecular weight for all molecule types. For the abundant proteins MetE, TufA, and CspC, and the less abundant GFP, we find the ratio of these relative viscosities, η_rel_^T^/η_rel_^R^, to be 3.6, 3.0, 3.2 and 2.5, respectively using the ‘full’ model; perhaps surprisingly, similar numbers are also obtained with the ‘steric’ model (Figure 3F). These computed ratios are in quite good agreement with the value of η_rel_^T^/η_rel_^R^ of 2.6±0.2 obtained from *in vitro* data for apomyoglobin diffusion in human serum albumin [28] (see Methods) and the value of η_rel_^T^/η_rel_^R^ of 2.1±0.3 reported for GFP in Chinese hamster ovary cells [29]; the lower value obtained in the latter case is consistent with the lower macromolecular concentration of the mammalian cytoplasm relative to that of *E. coli*.

### The thermodynamics of protein stability in the cytoplasm model

In addition to the simulations providing direct views of diffusive motions in the cytoplasm, snapshots extracted from the simulations offer an important opportunity to explore the thermodynamic consequences of the cytoplasm on macromolecular stability. Using a variant of Widom's ‘particle-insertion’ method [30], the free energy change that accompanies the insertion of a molecule into the cytoplasm can be rigorously computed by subjecting the molecule to millions of randomized placements (see Methods). We used this approach to compute the cytoplasm's effects on the folding equilibria of selected proteins by performing separate insertion calculations on their native state structures and on ensembles of 1000 unfolded structures generated by a sophisticated conformational sampling method [31]. We focused initially on the only two proteins for which experimental estimates of *thermodynamic* stability in the *E. coli* cytoplasm are available: (1) a construct of the λ-repressor N-terminal domain, λ_6-85_ [4], which has been found to have essentially identical stability *in vivo* and *in vitro*, and (2) the cellular retinoic acid binding protein [7],[32] (CRABP), which has been found to be thermodynamically destabilized *in vivo* relative to *in vitro*. Both of these findings – the latter in particular – are non-trivial results to capture since they are inexplicable in terms of conventional macromolecular crowding theory [2],[3],[7],[33],[34] (see below).

**Figure 4:**
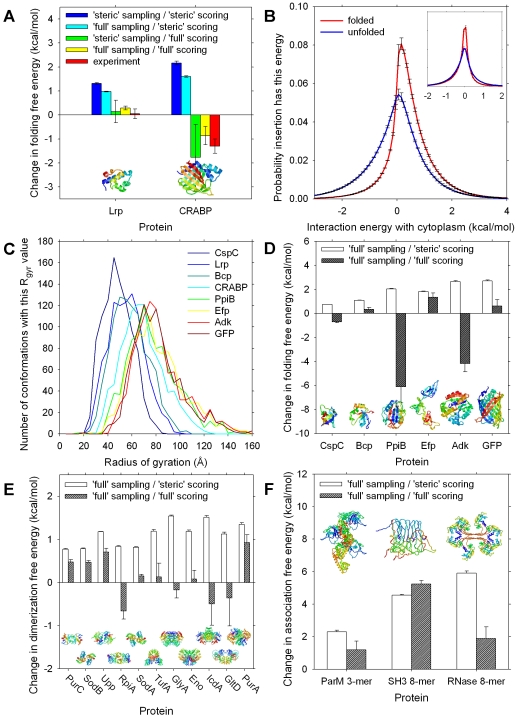
Thermodynamic effects of the cytoplasm model on protein folding and association equilibria. **A**. Computed stabilization of the folded state relative to the unfolded state for two experimentally-studied proteins; experimental data for Lrp (λ_6-85_) and CRABP taken from refs [4] and [32] respectively. ‘steric sampling’ indicates that insertions were performed on snapshots taken from a BD simulation performed with the ‘steric’ energy function; ‘steric scoring’ etc. indicates that the ‘steric’ energy function was used to calculate the cytoplasm-interaction energies, E_int_, of the inserted proteins. **B**. Histogram of interaction energies, E_int_, obtained for all non-clashing insertions of the folded and unfolded state conformations of CRABP with snapshots sampled from the ‘full’ model BD simulations; inset shows the same for λ_6-85_. **C**. Distribution of radius of gyration values for the 1000 unfolded conformations generated with the RCG software [31]; distributions are plotted in order of increasing molecular weight of the studied proteins. **D**. Same as A. but showing computed results for six other proteins, listed in order of increasing molecular weight. **E**. Computed stabilization of dimeric form relative to two separated monomers for eleven proteins, listed in order of increasing molecular weight. **F**. Computed stabilization of oligomeric form relative to separated monomers for three proteins.

We performed thermodynamic calculations under a total of four different scenarios. The first scenario that we examined involved taking cytoplasm snapshots sampled during the ‘steric’ BD simulations, and computing the cytoplasm-interaction energies of the folded and unfolded conformations with the same ‘steric’ energy model: this scenario corresponds to that considered in conventional models of macromolecular crowding effects [2]. In this case, the differences between the folding free energies *in vivo* and *in vitro* are computed to be +1.3±0.0 and +2.2±0.1 kcal/mol for λ_6-85_ and CRABP respectively (blue bars in Figure 4A), with the positive signs indicating that the folding free energies of both proteins are calculated to be more favorable *in vivo* than *in vitro*. When compared to the experimental values (red bars in Figure 4A), these results are in poor quantitative agreement for λ_6-85_ and are qualitatively wrong for CRABP. In a second scenario, we took cytoplasm snapshots sampled during the ‘full’ model BD simulations, but computed the cytoplasm-interaction energies of folded and unfolded conformations using the simpler ‘steric’ energy model. In this case, the differences between the folding free energies *in vivo* and *in vitro* are computed to be +1.0±0.0 and +1.6±0.0 kcal/mol for λ_6-85_ and CRABP respectively (cyan bars in Figure 4A). The smaller crowding effects obtained in this situation reflect the fact that during the ‘full’ BD simulations transient clustering of molecules creates bigger voids in the system; again however, these computed results are in poor quantitative agreement with experiment for λ_6-85_ and are in qualitative disagreement with experiment for CRABP.

A third scenario that we examined involved taking cytoplasm snapshots sampled during the ‘steric’ BD simulations and computing the cytoplasm-interaction energies with the ‘full’ energy model. In this case, the differences between the folding free energies *in vivo* and *in vitro* are computed to be +0.1±0.5 and −1.8±1.4 kcal/mol for λ_6-85_ and CRABP respectively (green bars in Figure 4A), both of which, notwithstanding the larger error bars, are in rather good agreement with the experimental results. Finally, we took cytoplasm snapshots sampled during the ‘full’ model BD simulations and computed the cytoplasm-interaction energies with the same ‘full’ energy model. In this fourth scenario – which on the basis of the diffusional properties described above would be hoped to provide the most realistic description (Figure 2A) – the computed changes in stability amount to +0.3±0.1 and −0.9±0.4 kcal/mol for λ_6-85_ and CRABP respectively (yellow bars in Figure 4A); again, these results are in close quantitative agreement with the experimental results. The overall picture that emerges, therefore, is that the experimental results cannot be reproduced, even qualitatively, when the ‘steric’ energy model is used to score the interactions between the folding protein and the cytoplasm environment, but they can be reproduced – and with a perhaps surprisingly high degree of quantitative accuracy – when the ‘full’ energy model is used in the particle-insertion calculations. Furthermore, the fact that similarly good results are obtained regardless of which energy model was used in the BD simulations suggests that, for such calculations, the method of sampling the cytoplasm's configurations is perhaps less important than the nature of the energy function used to describe the protein of interest's interaction with it.

Histograms of the computed interaction energies of the folded and unfolded state with the cytoplasm explain why the predictions of the ‘full’ model successfully reproduce experiment, and deviate so significantly from the predictions of the purely steric model: for both proteins, but especially so in the case of CRABP, the unfolded state conformations are computed to have somewhat more favorable energetic interactions with the cytoplasm than the folded state conformations (Figure 4B). The consequence is that while the excluded-volume (crowding) effect experienced by both proteins undoubtedly significantly stabilizes their folded states relative to their unfolded states (e.g. see the blue and cyan bars in Figure 4A), the effect is counterbalanced by the more favorable energetic interactions engaged in by the unfolded state conformations.

To explore the potential generality of this latter result, we performed identical calculations for a number of other monomeric proteins using snapshots taken from the ‘full’ model BD simulations; histograms illustrating the size distributions of the unfolded states of the tested proteins are shown in Figure 4C. The computed changes in their folding free energies are plotted in order of increasing molecular weight in Figure 4D. As before, when the ‘steric’ energy model is used to compute the cytoplasm-interaction energies the proteins' stabilities are computed to increase (white bars in Figure 4D); the computed stability changes scale broadly with the molecular weight of the protein, reflecting the greater relative difference between folded and unfolded state dimensions of larger proteins. In contrast, when the ‘full’ energy model is used to compute the cytoplasm-interaction energies, the molecular weight dependence is lost (dark grey bars in Figure 4D): some proteins are computed to be stabilized and others destabilized *in vivo* relative to *in vitro* (in no case however is the extent of destabilization sufficient to predict that the proteins will be predominantly unfolded *in vivo*). These results suggest that differences between the *in vitro* and *in vivo* thermodynamic stabilities will vary significantly with the identity of the protein.

### The thermodynamics of protein-protein interactions in the cytoplasm model

We performed similar calculations to explore the potential thermodynamic effects of immersion in the cytoplasm on a variety of protein-protein associations. For the formation of homo-dimeric complexes (Figure 4E), we again find that the excluded-volume crowding effect, which alone stabilizes dimers relative to separated monomers by on average 1.1±0.3 kcal/mol, is largely cancelled by the more favorable energetic interactions that the monomers form with the cytoplasm constituents: when the ‘full’ energy model is used the stabilization of the dimeric forms by the cytoplasm is computed to be, on average, only 0.1±0.3 kcal/mol. For the assembly of the trimeric nucleus [35] of the bacterial cytoskeletal protein ParM from three separated monomers, we find that the stabilization predicted with the ‘full’ energetic model is also significantly lower than that predicted from the crowding effect alone (Figure 4F); again, the smaller value appears more consistent with the close similarities between the polymerization behavior of ParM observed *in vitro* and *in vivo* [36]. Finally, we performed calculations on the assembly of two published (but putative) structural models of amyloid-like aggregates [37],[38], each formed by association of 8 monomer units (Figure 4F). For one of these two cases, the aggregation of an SH3 domain [37], we find that the use of the ‘full’ model predicts a slightly greater stabilization than that predicted solely on the basis of the crowding effect; the additional stabilization observed in this case results from the protein's interactions with the cytoplasm being dominated by repulsive electrostatic interactions, which, on average, are diminished in the aggregated state (see Figure S3).

## Discussion

Developing working computational models of intracellular environments is one potential route to understanding differences between biomolecular behavior observed *in vitro* and *in vivo*. The simulations and calculations described here represent the first attempt to build such a model for the bacterial cytoplasm using atomically detailed structures of the constituent molecules, and represent the first attempt to directly model the consequences of immersion in the cytoplasm on the thermodynamics of protein stability and protein-protein interactions. It is worth noting that these innovations have been made possible in large part due to the immense progress made by the structural biology community in recent years: in constructing our model it was a major surprise to us to find that, of the 50 most abundant cytoplasmic *E. coli* proteins identified in the study of Link *et al.* [12], it was possible to produce complete or near-complete structural models for more than 45 (see Supporting Information). Since large-scale structural genomics initiatives continue to map out the structural proteomes of organisms with ever increasing detail [39] it will be possible to make future generations of cytoplasm models even more compositionally complete.

Before considering the strengths and weaknesses of the present model, and the implications of the results reported here, it is important to reiterate that at least two other cytoplasm models have already been reported in the literature. The first such model was described by Bicout and Field [10] some thirteen years ago. Owing to the comparative paucity of both structural information and computer power then available, the model was restricted to only three types of macromolecule, each of which was modeled as a sphere: their modeled system contained 12 ribosomes, 188 copies of a generic protein of molecular weight 160kDa, and 136 tRNAs. Langevin dynamic simulations were used to model behavior over a timescale of 7.5µs, and four different electrostatic approximations were investigated in an attempt to cover a range of possible simplified descriptions of the ribosome's electrostatic properties. With all four models, the long-time translational diffusion coefficient of the modeled protein was slowed by only ∼40% relative to its infinite-dilution value. Since their work pre-dated the first reports of D_trans_ values measured *in vivo*, Bicout and Field could not know at the time that this simulated diffusion was too fast relative to experiment; they were therefore not in a position to more fully calibrate their model. Despite this issue, it should be clear to readers that the work of Bicout and Field was far ahead of its time. It should also be apparent that, like the influential work of Goodsell [20], it was a direct inspiration for the work reported here.

A second and much more recent model for the bacterial cytoplasm has been developed by Ellison and co-workers [11]. Relative to Bicout and Field's work, the model of Ridgway, Broderick *et al.* provides an enormous step forward in terms of compositional complexity: >100 different types of proteins are represented, and thanks to the availability of the authors' own proteomic data [40], are present in copy numbers that are likely to much more closely reflect their relative abundances *in vivo*. On the other hand, all macromolecules are treated as spheres, and intermolecular interactions are assumed to be purely steric in nature. In addition, the actual modeling of motion is somewhat simplified: particles take steps of uniform length in randomly chosen directions, with the steps being accepted only if no collision – or reaction – with a neighboring molecule occurs. While somewhat approximate, this approach has the significant advantage of allowing reactive events to be rapidly modeled, making the simulation model applicable to a more general set of problems than that considered here. The resulting model of the cytoplasm was used to investigate the effects of crowding on the translational diffusion of macromolecules and on the rate of the diffusion-limited association of the barnase-barstar protein-protein complex. As noted by the authors, the diffusional simulations produced only a two-fold decrease in the translational diffusion coefficients of GFP-like molecules, suggesting, in common with the results reported here, that (steric) crowding effects alone are insufficient to explain the ∼10-fold slowed diffusion of GFP observed *in vivo*.

Relative to these two previous cytoplasm models, therefore, the present approach offers a significant increase in both structural and energetic complexity: all macromolecules are modeled in atomic detail and interact with one another via an energetic model that accounts for the two major types of interaction that drive protein-protein associations (i.e. electrostatic and hydrophobic interactions). It does so, of course, at very significant computational expense: each of the simulations performed with our ‘full’ energy model required more than a year of clock-time to complete. But even with its associated expense it should not be thought that the present model represents the pinnacle of sophistication in terms of its description of reality. Leaving aside the fact that the model is incomplete in terms of the types of macromolecules (and small molecules) that it includes, there are several key assumptions of the modeling that are both important to stress and which represent obvious candidates to address further in future work.

A first simplification of the present approach, and one shared by the previous models described above, is that all macromolecules have here been treated as rigid bodies. This simplification has two consequences. First, it immediately precludes us from making any meaningful attempt to simulate the (presumably very interesting) diffusive behavior of highly flexible macromolecules such as mRNAs and intrinsically unstructured proteins. While this is undoubtedly a limitation, it is to be noted that in terms of their contributions to the overall mass content of the cytoplasm, such molecules play a comparatively minor role *relative* to that played by the folded, globular macromolecules examined here [10]. It is also to be noted that there are currently very serious technical obstacles to be overcome if the diffusive behavior of flexible macromolecules is to be simulated with any degree of realism: we have shown recently, for example, that the inclusion of hydrodynamic interactions (HI), which are computationally very expensive to compute, is essential if flexible protein models are to adequately reproduce translational and rotational diffusion [41]. A second consequence of the rigidity of the present model is that it is not immediately suited to describing conformational changes that might potentially occur in highly crowded conditions, and for which interesting experimental and simulation results have recently been reported [42],[43]. As shown in the second part of this paper however, this limitation can be overcome, at least for the purposes of calculating thermodynamic effects, by the use of particle-insertion calculations. In fact, the use of such an approach has enabled us to explicitly evaluate the cytoplasm's thermodynamic consequences on both folding and association equilibria, something that would currently be prohibitively expensive to achieve through the direct dynamic simulation of flexible protein models.

A second, but not unrelated simplification adopted in the present approach concerns the energy model used to describe intermolecular interactions. On the one hand, the model is comparatively sophisticated in that it includes descriptions of electrostatic and hydrophobic interactions, and models both at an atomic, or near-atomic level of resolution: in this respect it is a clear improvement over previous models used to simulate the cytoplasm. On the other hand, the model assumes that electrostatic desolvation effects can be neglected (which may lead to an overestimation of the strength of electrostatic interactions; [44]) and treats hydrophobic interactions as pairwise additive [45],[46] and of equal strength for aliphatic and aromatic groups. We assume that the effects of these missing features are at least partly subsumed, in an implicit fashion, within our single hydrophobic parameter, ε. For this reason, we should be careful not to attach too much importance to the absolute value of ε found here (0.285 kcal/mol): it is, nevertheless, encouraging that it is very similar to the range of values that we previously obtained [19] when modeling the thermodynamics of simple dilute protein solutions (0.22–0.28 kcal/mol). This is perhaps especially notable given the enormous difference between the protein concentration studied here (275mg/ml) and that studied in the previous work (10mg/ml).

In future, it should be possible to increase the sophistication of the energy model without incurring an exorbitant additional computational cost: if one stays with a rigid-body approach, for example, a number of grid-based methods might be used that allow electrostatic desolvation [44] and/or hydrophobic interactions [47]–[50] to be rapidly calculated. It should be remembered, however, that a more complicated functional form will not necessarily lead to better results, and that, at least for now, it is highly likely that some degree of empirical adjustment of energy terms will be required in order to reproduce experimental behavior. This will be especially true if the intention is to use a similar model to explore, for example, macromolecular crowding effects on *specific* protein-protein interactions: despite significant advances, no current computational method is capable of accurately predicting the strength or geometry of specific protein-protein interactions with any generality [51]. To model such situations, therefore, it may be necessary to supplement the energy model with additional short-range forces to drive the formation of known intermolecular contacts, in the same way that such terms (commonly known as Gō-potentials; [52]–[54]) are often used in the modeling of protein folding processes; an alternative might simply be to use different ε values for different protein-protein interactions.

A third limitation of the present model concerns its very simplified description of macromolecular hydrodynamics. In particular, while the *basic* hydrodynamic properties of all macromolecules (i.e. their translational and rotational diffusion coefficients at infinite dilution) are properly accounted for, the BD simulations reported here do not allow for the presence of hydrodynamic interactions (HI) *between* macromolecules; again this is true also of the two previously reported cytoplasm models [10],[11]. The immense expense associated with HI calculations remains a major stumbling block to their inclusion in large-scale simulations [55] and a number of attempts have therefore been made to accelerate their computation (see, e.g. [56],[57] for very recent and potentially important examples). This expense would be further increased in the present case if, as would in principle be necessary, an Ewald summation technique was used to properly account for HI in periodic boundary conditions [58].

While simply stating that HI are expensive to calculate does not constitute a compelling reason for leaving them out of the simulations, it is pertinent to note that the omission of HI seems unlikely to be the cause of the gross overestimation of the diffusion coefficient of GFP obtained with the ‘steric’ energy model (Figure 2A). It is certainly true, as noted elsewhere [18], that for hard-sphere-like colloidal particles – where the interactions between particles are extremely short-range – theoretical work has established that the inclusion of HI should cause decreases in D_trans_ values over both short [59] and long timescales [60],[61]. Such decreases are, however, unlikely to bridge the ∼5-fold gap necessary to bring the ‘steric’ energy model behavior into quantitative agreement with experiment: in an interesting recent simulation study, for example, it was found that an approximate description of HI in crowded hard-sphere solutions resulted in only a ∼40% additional decrease in the diffusion coefficient relative to simulations without any description of HI [62]. In addition, it is also to be noted that for colloidal particles with long-range repulsive electrostatic interactions, theory indicates that the inclusion of HI causes modest *increases* in D_trans_ values at both short [63],[64] and long timescales [64],[65]. Since the current model has macromolecules interacting with each other not only by short-range steric forces and long-range repulsive electrostatic forces, but also by short-range attractive interactions between exposed hydrophobic residues it is difficult to predict the effects that the inclusion of HI might ultimately cause, other than to say that we think they may be *comparatively* modest. In keeping with the caveat given above about our energy model, however, we clearly must leave open the possibility that the hydrophobic parameter, ε, is also, in part, serving as an implicit correction for the omission of HI from the simulations.

Having produced in the preceding paragraphs a litany of shortcomings of the model one might be tempted to view it as so fundamentally limited that its practical utility is in doubt. Perhaps the strongest argument against such a view comes from the results of the particle-insertion calculations aimed at computing the thermodynamics of protein folding *in vivo* (Figure 4A). It is important to note that these thermodynamic calculations should be considered *bona fide* predictions of the simulation model since it was calibrated to reproduce a quite different experimental observable, i.e. the translational diffusion coefficient of GFP. Because of this, we can rule out the possibility that the calibration of the model predisposes it to trivially reproduce experimental protein stability effects. To our knowledge, the calculated results reported here with our ‘full’ energy model are the first to provide a quantitative rationalization of the experimental observation that CRABP is destabilized *in vivo* (relative to *in vitro*) and that λ_6-85_'s relative stability is essentially unchanged. As noted earlier, the experimental CRABP result is inexplicable with conventional macromolecular crowding theory (as exemplified by the results obtained here when the ‘steric’ energy model is used in the particle-insertion calculations) since the dimensions of its unfolded state are greater than those of its native state. Use of the ‘full’ energy model, on the other hand, produces results in close agreement with experiment because it explicitly allows for the two states of the protein to engage in differential, favorable energetic interactions with the rest of the constituents of the cytoplasm. Interestingly, good results are obtained when the ‘full’ energy model is used in the particle-insertion calculations regardless of whether the cytoplasm snapshots were sampled from the ‘steric’ BD simulations or sampled from the ‘full’ BD simulations. Although the most internally consistent approach is obviously to use the same energy model in both the BD simulations and the particle-insertion calculations, the fact that good results can apparently also be obtained using snapshots from the ‘steric’ BD simulations is intriguing since such simulations are much faster to conduct than those using the ‘full’ energy model. Our model's predicted effects on the folding free energies of the six other proteins investigated (Figure 4D) await experimental testing of course, but regardless of how quantitatively accurate such predictions might eventually turn out to be we feel reasonably confident in suggesting that future attempts to understand a protein's folding thermodynamics *in vivo* will need to describe its interactions with the cytoplasm with more realism than is provided by simple steric interactions.

Other findings from the simulations, while probably more difficult to directly test experimentally, provide examples of the kinds of new information that can be obtained from simulation approaches that attempt to model intracellular environments. Examples include the observation that the immediate neighbors of individual proteins exchange rapidly on a microsecond timescale – even for the very largest macromolecules – and that diffusion is transiently anomalous even on a sub-nanosecond timescale. The latter observation is especially interesting given the current interest in anomalous subdiffusion as an efficient mechanism of search and association in physiological situations [8],[66]. Finally, one might also point to the fact that the simulation model correctly reproduces the cytoplasm's relative translational and rotational viscosities as an important favorable result since differential effects on translational and rotational motion appear to have interesting effects on protein-protein association rates in crowded solutions [67]–[69]. It should be remembered, however, that a similarly good reproduction of the relative translational and rotational viscosities is also obtained with the otherwise poorly performing ‘steric’ energy model.

An examination of all of the dynamic and thermodynamic results described above shows, we think, that it is possible to leverage the existing structural biology and quantitative proteomic data to produce a meaningful, working molecular model of the bacterial cytoplasm. The actual simulation model used here has a number of limitations, of course, but continuing increases in computer power and/or the development of faster simulation methodologies, will likely allow many of these drawbacks to be eliminated in the not too distant future. Given the continuing progress in the fields of structural biology and quantitative proteomics it is likely that the same basic approach might be used to model other intracellular environments.

## Methods

### Selection of the constituents for the cytoplasm model

When this work was initiated, the only large-scale quantitative study of the *E. coli* proteome was that reported by Link *et al.* [12] who experimentally measured levels of >200 of the most abundant proteins present in *E. coli*. A number of other quantitative proteomic studies of *E. coli* have since been reported [40],[70],[71], and, since this work was completed, quantitative estimates of metabolite concentrations have also become available [72]. Restrictions on computer memory (4GB of RAM for all servers used) meant that the total number of different *types* of macromolecules that could be realistically modeled was limited to 51: these would be 50 types of *E. coli* macromolecule plus the Green Fluorescent Protein (GFP). Although including only 50 different types of macromolecules means that the model underestimates the structural diversity of the cytoplasm, it is important to note that the macromolecules selected for inclusion account for 85% (by number of protein chains) and 86% (by mass) of *all* the cytoplasmic proteins quantified and identified in Table 4 of Link *et al.* [12].

Of the 50 types of *E. coli* macromolecules to be included in the model, 45 would be proteins. These were selected by working down the list identified by Link *et al.* in order of decreasing abundance, selecting only those proteins (a) for which high-resolution structures were then available in the Protein Data Bank [13] (PDB) or for which reasonable homology models could be constructed (see below), and (b) for which the cytoplasm was unambiguously identified as the major cellular location in the EcoCyc [73] and/or CCDB [74] databases. A full list of all potentially cytoplasmic proteins identified and quantified in Table 4 of Link *et al.* (under minimal media conditions), arranged in decreasing order of chain-abundance, is shown in Table S1; asterisks in the columns headed ‘Mod.’ denote those proteins included in our cytoplasm model. It is an indication of the tremendous coverage of the structural proteome that has been achieved by the structural biology community that we were able to obtain, or build, reasonable structural models for *all* of the 30 most abundant cytoplasmic proteins identified by Link *et al.* [12]. In addition to the 45 different types of proteins, 5 types of macromolecule were RNAs or RNA-protein complexes: these were the two ribosomal subunits (50S and 30S), and three typical tRNAs for which structures were available: (tRNA-Gln, tRNA-Phe and tRNA-Cys). It is to be noted that we did not model complete (translating) 70S ribosomes owing (a) to the inherent difficulties in modeling the flexible mRNA, and (b) to the absence – at the time this work was begun – of a three-dimensional structure showing the arrangement of multiple 70S ribosomes in a polyribosome [75].

The total number of molecules in the simulations was set to 1008 (eight copies of GFP and 1000 *E. coli* macromolecules). This number was chosen so that the eventual assembled cytoplasm model would be large enough to provide a reasonable representation of the environment while still allowing simulations of up to 20µs to be performed (albeit over the course of more than a year clock-time). The linear dimensions of the final modeled system (808.4Å in each of the x, y and z directions) correspond to approximately one-twelfth of the diameter of a typical *E. coli* cell [76]. A summary of the macromolecules selected, their subunit compositions, the PDB codes of their originating structures, and the degree of sequence coverage achieved by the structural models, is presented in Table S2. Using composition estimates provided by Neidhardt *et al.* [76] as a guide, we set the total concentration of macromolecules in the model (excluding the ‘tracer’ GFP) to 275 g/l; this is slightly on the low side of the rough values of 300–340 g/l estimated independently by Zimmerman and Trach [77]. Of this, 55g/l (i.e. 20% of the total) is contributed by RNA, with 15% of the RNA dry weight contribution being made by tRNA and the remainder being made by ribosomal RNA [76]. mRNA, which accounts for only ∼4% of the total dry weight of RNA in the cell, is omitted from the present model. The remaining 219g/l (i.e. 80%) of the model is contributed by proteins; this percentage is deliberately set somewhat higher than the 55% contribution to the dry weight of the entire cell estimated by Neidhardt *et al.* [76] in order to compensate for the missing volume of components that are not explicitly represented in the model (DNA, mRNA, lipid, lipopolysaccharide, murein, and glycogen). If one takes the specific volumes of proteins and RNA to be 0.73ml/g and 0.58ml/g respectively [77], the total volume fraction occupied by macromolecules in the model is 0.19; if instead, an ‘effective’ specific volume of macromolecules suggested by Zimmerman and Trach is used [77] (1.0ml/g), the total volume fraction occupied by the macromolecules in the model amounts to 0.27.

### Preparation of the macromolecular structures for simulation

Structures for all selected proteins were identified by performing a BLAST search [78] of the protein's FASTA sequence (as reported in the EcoCyc database) against the entire PDB and selecting the structure with the closest identity to the query sequence using the program BioEdit [79]. The quaternary structure of each selected structure was determined using the PQS web server [80] and was verified, where possible, with the EcoCyc database; it should be noted that correct identification of a protein's quaternary structure is a non-trivial undertaking, and the PQS predictions are unlikely to be 100% reliable [80],[81]. Homology modeling was used for all proteins for which either no *E. coli* structure was directly available in the PDB, or for which a significantly greater coverage of the sequence could be obtained through the use of a non-*E. coli* structure. All homology modeling was performed using the SWISS-MODEL web server [82] via the so-called “First Approach mode”; for oligomeric proteins each individual chain was homology-modeled independently.

Any sidechains missing from a structure were built in using the molecular modeling program WHATIF [83]. Hydrogens were then added, and partial charges and radii were assigned to atoms using the program PDB2PQR [84]. For proteins, partial charges and atomic radii were taken directly from the PARSE parameter set [85]. For nucleic acids, which are not represented in the PARSE parameter set, partial charges were instead assigned from the CHARMM23 parameter set [86]; partial charges for the modified bases of tRNAs, such as pseudouridine, were assigned based on similarity to functional groups already represented in the parameter sets. The protonation states of all protein ionizable residues were assigned using the fast empirical algorithm PropKa [87]; for these calculations, the pH was set to 7.6, the estimated pH of the *E. coli* cytoplasm [76]. With each structure complete, infinite-dilution translational and rotational diffusion coefficients – which are necessary input parameters for BD simulations [14] – were calculated with the program HYDROPRO [88] using default parameters. For the latter calculations we assumed a solvent viscosity, η, of 0.89cP, which corresponds to the viscosity of pure water at 25°C; given that the most recent estimate of the total metabolite concentration in the *E. coli* cytoplasm is ∼300mM [72] we do not anticipate, based on what we currently know, that the viscosity of the solvent environment will be hugely altered from the pure water value.

The final stage of preparation for each molecule involved the calculation of electrostatic potential grids; these were computed in all cases by using the APBS software [89] to solve the non-linear Poisson-Boltzmann (PB) equation [90]. As in our previous BD study of single-component protein solutions [19], two cubic electrostatic potential grids were computed for each type of macromolecule: (a) a *comparatively* fine grid, of spacing 2Å, with dimensions sufficient to encompass a 20Å shell around the macromolecular surface, and (b) a coarse, long-range grid, of spacing 4Å, that extends at least 50% further in each direction than the smaller grid. The use of a 2Å grid spacing for the higher resolution grids, rather than the 1Å grid spacing used in our previous simulations [19], was necessary in order to fit all potential grids into the available 4GB of RAM. This spacing is, however, sufficiently detailed that at least two grid points always intervene between interacting atoms even when they are at the closest possible separation distance (4.5Å); significant numerical instabilities in the calculated electrostatic forces do not, therefore, arise. In all PB calculations the solvent dielectric was set to 78.0 and the internal dielectric of the macromolecule was set to 12.0, with the boundary between the two being determined by the cubic-spline surface [91] implemented in APBS [89]. Use of an internal dielectric of 12.0 is intended to provide a simple, averaged description of the different dielectric responses of macromolecular interiors and exteriors [19],[92],[93]. The ionic strength in all PB calculations was set to 150mM. With the electrostatic potentials in hand, ‘effective charges’ were computed for each molecule type using the procedure established by Gabdoulline & Wade [94],[95]. Finally, as in our previous work [19], simulations were accelerated by retaining, in addition to the effective charges, only those non-hydrogen atoms that were solvent-exposed: these atoms were identified using the ACC tool within APBS [89], with a 4Å solvent probe.

### Brownian dynamics simulation protocol

The BD software used for the simulations is an extension of the methodology developed and tested in our previous work on pure protein solutions [19]. Modifications were made to the software to improve memory usage so that 102 electrostatic potential grids could be simultaneously held in memory; in addition, toward the end of this study, loop-level parallelization of a number of key loops was implemented with OpenMP (http://www.openmp.org) to accelerate computations by a factor of ∼4.

All simulations were performed under periodic boundary conditions [96] in a cubic cell with edges of 808.4Å. The initial configuration of each system had eight GFP molecules evenly positioned at the center of the eight octants of the cell; all other macromolecules were initially positioned by performing random translations and rotations within the cell subject to the requirement that there was at least a 10Å separation between the surfaces of all neighbors. Three independent configurations were generated in this way by use of different random seeds; views of each system before and after 15µs of simulation are shown in Fig. S1. As in our previous work, BD simulations were conducted using the Ermak-McCammon algorithm [97] with a time step of 2.5ps, with additional algorithmic measures being taken to ensure that no atom-atom distances at the completion of each timestep were less than 4.5Å. For subsequent analysis of the simulations, the 3D translational vector and the 3×3 rotational matrix necessary to specify the position of each macromolecule were recorded every 100ps.

The form of the energy model used to describe intermolecular interactions was identical to that used in our previous work [19]: the effective charge method [94] was used to calculate electrostatic interactions, and a Lennard-Jones potential (comprising 1/r^12^ and 1/r^6^ terms) was used to provide a simple combined description of steric, van der Waals and hydrophobic interactions. To accelerate the simulations, the combined non-electrostatic interactions were computed only between atom pairs separated by less than 12Å; a list of all such pairs was continually updated every 40 timesteps (i.e. every 100ps). As in our previous work, we treated the strength of these non-electrostatic interactions, which are determined by the well-depth, ε_LJ_, of the Lennard-Jones potential, as the *only* adjustable parameter of the model. In order to determine the best setting, three independent BD simulations of at least 6µs duration were performed with each of the following ε_LJ_ values: 0.190, 0.285, 0.3325 and 0.380 kcal/mol. Finally, for comparison purposes, two additional sets of three BD simulations were also performed: these were (a) simulations in which the only the repulsive (1/r^12^-dependent) steric interactions operated (these are the ‘steric’ simulations discussed in the main text) and (b) simulations in which only steric plus electrostatic interactions acted.

### Analysis – translational diffusion coefficients

The effective translational diffusion coefficients, D_trans_, of molecules were calculated from the simulations using the Einstein equation:
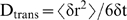
(1)where < δr^2^ > is the mean-squared distance traveled by the molecular center of mass in the observation interval, δt; all D_trans_ values reported in Results are mean values for each molecule type averaged over the number of copies of each type. In cases of ‘normal’ diffusion, the computed D_trans_ values are independent of δt; in certain cases of diffusion *in vivo* and *in vitro* however, anomalous sub-diffusion is observed [8], [21]–[23],[66]; in such cases, the apparent D_trans_ value is dependent on δt, decreasing with increasing δt. A common way of describing anomalous diffusion involves writing it in the form:
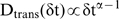
(2)where the apparent translational diffusion coefficient D_trans_ is now written to indicate that it depends on the observation interval and α is the so-called anomalous diffusion (anomality) exponent; α = 1 corresponds to normal diffusion since it leads to D_trans_ being independent of δt, and α<1 indicates anomalous (sub)diffusion. Taking logarithms and differentiating with respect to log (δt) allows us to write:
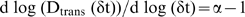
(3)This enables us to obtain α by numerically differentiating D_trans_ values computed over a range of δt values; in practice we computed D_trans_ at δt values of 100, 200, 300, 600, 1000, … ps, and obtained α at the logarithmic mid-point, δt_mid_, of these time-intervals, δt_mid_ = 141, 245, 424, … ps.

Plots of α versus log (δt_mid_) for macromolecules simulated with both the ‘steric’ and ‘full’ energy models all indicated that α itself was dependent on δt_mid_, thus signifying that diffusion was *transiently* anomalous. To our knowledge, there is no explicitly derived functional form that describes the expected dependence of α on δt for transient anomalous diffusion. We found however that the data fit well to the following empirical functional form (see Fig. 3B):
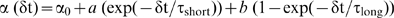
(4)where α_0_ is a constant, *a* and *b* are parameters that describe the amplitude of the δt-dependent changes to α, and τ_short_ and τ_long_ are, respectively, the timescales over which α first decreases, and then returns to one, with increasing δt. Plots of α versus δt for all molecule types were fit to the above functional form with SigmaPlot [98]: fits were performed using all datapoints from the shortest δt_mid_ value up to the first datapoint that had a percent error exceeding ∼25% (obtained by comparing the α values computed from the three independent BD simulations), or that deviated qualitatively from the trend. To ensure that the latter criterion did not drastically affect the results, the fits were repeated retaining even those datapoints that qualitatively deviated; essentially the same behavior was obtained but with slightly greater values of τ_long_. Regressed values of τ_short_ and τ_long_ are plotted versus molecular weight for all molecule types in Figs. S4 and S5 respectively.

Having fit a function to the observed dependence of α on δt, it was numerically integrated to obtain an extrapolated, asymptotic long-time D_trans_ value using the D_trans_ value at δt = 100ps as the starting point for the integration. The quality of fits of the integrated D_trans_ values (for the most abundant proteins) is indicated by the solid lines in Fig. 3A.

### Analysis – rotational diffusion coefficients

Effective rotational diffusion coefficients were computed from the time-dependent behavior of the 3×3 rotational matrix recorded every 100ps for every molecule during the simulations. For each of the three rotational axes, an autocorrelation function, θ (δt), was calculated as:
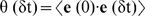
(5)where **e** (0) and **e** (δt) are unit vectors pointing along one of the rotational axes at time t = 0 and t = δt respectively, and the brackets indicate an average over all possible initial timepoints; the three computed autocorrelation functions were averaged to give a single decay function consistent with the isotropic rotation that we assumed for all molecule types at infinite dilution. Since the resulting averaged autocorrelation function for the ‘full’ energy model did not fit well to a single-exponential decay, and given that *translational* diffusion was clearly transiently anomalous, we decided to use the following functional form proposed recently for transiently anomalous *rotational* diffusion [27]:
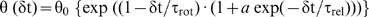
(6)where θ_0_ is the value of the autocorrelation function at δt = 0 (always 1), *a* is a parameter, τ_rot_ is a long-time rotational correlation time (which dominates as δt→∞), and τ_rel_ is the timescale over which a faster, short-time rotational relaxation gives way to the slower rotation characterized by τ_rot_. The above functional form was fit to computed values of θ for each molecule type over a range of δt values up to 1µs; the r^2^ values for these fits were all in excess of 0.999. An example of such fits for the most abundant proteins is shown in Fig. S6. The long-time rotational diffusion coefficient, D^L^_rot_, is then obtained using the relationship:
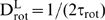
(7)and the short-time rotational diffusion coefficient, D^S^_rot_, is obtained from [27]:
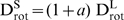
(8)The computed ratios D^L^_rot_/D^0^_rot_ and D^S^_rot_/D^0^_rot_ obtained with the ‘full’ energy model are plotted for all molecule types versus their molecular weights in Fig. S7; a plot of the parameter *a* versus molecular weight shows no obvious relationship (not shown).

### Analysis – literature estimates of relative translational and rotational viscosities

Comparison of the simulated translational and rotational diffusion coefficients with the infinite-dilution values that are input parameters for the simulations provides an indication of the relative viscosities experienced during the two types of motion. From studies of GFP diffusion in Chinese hamster ovary cells, the Verkman group reports [29] a relative viscosity experienced by translational motion, η_rel_^T^ = 3.2±0.2, and a relative viscosity experienced by rotational motion, η_rel_^R^ = 1.5±0.1. Combining these numbers gives a ratio, η_rel_^T^/η_rel_^R^ of 2.1±0.3, indicating that the effective relative viscosity experienced by translational motion is roughly twice that experienced by rotational motion in mammalian cells.

A second estimate of the η_rel_^T^/η_rel_^R^ ratio can be obtained from the work of Zorrilla *et al.* [28],[99]: these authors have reported measurements of the translational diffusion coefficients of apomyoglobin (17kDa) using fluorescence correlation spectroscopy (FCS) measurements [28] and have compared them with rotational diffusion coefficients that they had previously measured [99] for the same system using time-resolved fluorescence depolarization experiments. They report measurements for two different background proteins, RNaseA and human serum albumin (HSA); we focus on the data reported for the latter since its molecular weight (67kDa) is much closer to the number-averaged molecular weight of the macromolecules in our cytoplasm model (87kDa), than is the molecular weight of RNaseA (14kDa).

The data reported by Zorrilla *et al.* are expressed relative to the macroscopic viscosity, η_m_, of the protein solution (measured with an Ostwald viscometer). They report that η_m_ fits to the following functional form, η_m_ = η_0_ exp (A*c*/(1−B*c*)), where η_0_ is the viscosity of pure water, *c* is the background protein's concentration in mg/ml, and A and B are background-dependent constants: A = 2.7×10^−3^ ml/mg and B = 1.3×10^−3^ ml/mg for HSA [99]. Using these values we obtain a macroscopic viscosity for a 275 mg/ml HSA solution of 3.155 η_0_. Using the data given in Table 2 of ref. 49, the effective viscosity experienced by the *translational* motion of apomyoglobin in HSA is expressed as η_rel_^T^ = (η_m_/η_0_)^1.28^, which from above means that we can write η_rel_^T^ = 3.155^1.28^ = 4.35; following similar calculations the effective viscosity experienced by the *rotational* motion is η_rel_^R^ = (η_m_/η_0_)^0.44^ = 3.155^0.44^ = 1.66. Together, these numbers translate into a value of η_rel_^T^/η_rel_^R^ of 2.6±0.2.

As noted in the main text, we find that *both* the translational and rotational diffusion coefficients of molecules vary with the time interval, δt, over which diffusion is observed. While the observation of this transient anomalous diffusion is significant in its own right it takes on added significance when comparing the relative viscosities experienced by translational and rotational motion. This is because the timescales over which the two types of experiments are conducted are quite different: translational diffusion coefficients are obtained from FCS experiments by fitting to an autocorrelation function over a timescale extending from microseconds to seconds [21],[22],[66] while rotational diffusion coefficients are obtained from fits to data obtained over a nanosecond timescale [28],[29]. We therefore compare the experimentally derived relative viscosities quoted above with diffusion coefficients computed from the BD simulations on the same timescales, i.e. we compare with the ratio of the long-time translational diffusion coefficient D^L^_trans_ and the short-time rotational diffusion coefficient, D^S^_rot_ (see Fig. 3F).

### Analysis – monitoring of intermolecular contacts

The intermolecular contacts engaged in by each molecule were recorded every 100ps during the BD simulations and subsequently analyzed to determine: (a) the average number of neighbors of each molecule type at any given time, (b) the number of unique neighbors encountered by each molecule type during the course of the entire simulations, and (c) the rate of dissociation of intermolecular interactions. The definition of ‘neighbor’ was kept somewhat loose in order to detect all molecules in the immediate environment of the molecule being probed: molecules were assigned as neighbors if any of their atoms were within ∼12Å of each other. The rates at which the neighbors of a particular molecule dissociated were obtained from plots of the fraction of its neighbors, initially present at t = 0, that remained after some time t = δt, averaged over all possible initial timepoints. In order to obtain the characteristic neighbor-decay rate for each particular type of molecule, such plots were averaged over all molecules of that type. The resulting plots are found to follow biexponential kinetics: (a) a very fast decay process (τ_fast_) that typically has an amplitude of ∼0.7 and is due to loss of neighbors that interact only peripherally with the molecule of interest, and (b) a slower decay process (τ_slow_) that has an average amplitude of ∼0.3 and is due to loss of those neighbors that form *bona fide* intermolecular contacts. Typical fits for these data are shown in Fig. S8.

### Method for calculating thermodynamics in the cytoplasm

The effects of immersion in the cytoplasm on the thermodynamics of protein folding and protein-protein association were computed using the particle insertion technique first outlined by Widom [30]. For small perturbations, the free energy change, ΔG, for transferring a molecule from an environment free of any interacting macromolecules to the cytoplasm environment can be rigorously expressed as:
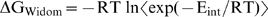
(9)where E_int_ is the interaction energy of the molecule with the constituents of the cytoplasm, R is the Gas constant, T is the temperature, and the brackets indicate an average over randomly selected insertion positions and configurations of the cytoplasm environment. In order to assess the likely effects of the cytoplasm on a thermodynamic process (such as protein folding) therefore, separate particle-insertion calculations are required for both the initial state (e.g. unfolded protein) and the final state (e.g. folded protein). Such calculations give the free energy changes for the vertical processes in the thermodynamic cycle shown below:
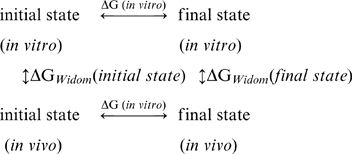
(10)Since free energy is a state function, the difference between the free energy changes of the horizontal processes is equal to the difference between the free energy changes of the vertical processes. We can therefore write the difference between the free energy change for the process *in vivo* and *in vitro*, ΔΔG, as:
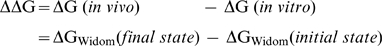
(11)The effect of the cytoplasm on the free energy change for a process can therefore be calculated without needing to know the actual value of the free energy change for the process *in vitro*. A conceptually similar but different approach to computing thermodynamics in crowded solutions has recently been outlined by Zhou and co-workers [100]. Code for performing particle-insertion calculations was generated by modifying the existing BD simulation program; prior to performing large-scale explorations of protein folding and association thermodynamics, the code's correctness was first checked by comparing its predictions for the free energy cost of placing a sphere into a solution of spheres with the corresponding predictions of scaled particle theory [101],[102].

### Cytoplasm effects on protein folding equilibria

Calculations of the cytoplasm's thermodynamic effects initially focused on protein folding equilibria. In addition to calculating the folding thermodynamics of six proteins already present in the cytoplasm model (Adk, Bcp, CspC, Efp, GFP and PpiB), we examined two other proteins that have been subject to direct experimental study *in vivo*: these were the 80-residue λ_6-85_ construct studied experimentally by Ghaemmaghami and Oas [4] and the 136-residue cellular retinoic acid binding protein (CRABP) investigated by Ignatova, Gierasch and co-workers [7],[32]. The structure of the folded state of λ_6-85_ was taken from its crystal structure in complex with operator DNA (pdbcode: 1LMB [103]); the G46A & G48A mutations present in the experimental construct were made using the rotamer-sampling method SCWRL3 [104]. The structure of the folded state of CRABP (pdbcode: 1CBI [105]) was altered to include the R131Q mutation used in the experimental construct [7], but in the absence of direct structural information no attempt was made to model the experimentally-incorporated fluorophore.

The unfolded states of all eight proteins were modeled as ensembles of 1000 unfolded conformations generated using the conformational sampling method developed by the Sosnick group [31]; the code was kindly made available by Dr. Abhishek Jha. This method has been shown to produce models with dimensions in good agreement with experimental estimates [31]. Prior to calculations, the structures of all conformations were completed by adding sidechains with SCWRL3 [104] and by adding hydrogens with the PDBTOPQR utility [84] of APBS [89]. In order to ensure consistency between the BD simulations and the Widom particle-insertion calculations, effective charges and electrostatic potential grids were calculated for all conformations (both folded and unfolded) using the exact same protocol employed with the rigid protein models of the cytoplasm model (see above).

For each protein, a large number of random trial positions were attempted with both the single, folded state structure and the 1000 unfolded state conformations; each trial consisted of a different randomly selected translation and rotation. For the folded state structure, a total of 25 million trials were attempted; for the unfolded state, 250,000 trials were attempted for each of the 1000 conformations (to give a total of 250 million trials for each cytoplasm ‘snapshot’ studied). For each trial position, the interaction energy of the protein with the surrounding cytoplasm was calculated with (a) the ‘full’ energetic model, which includes electrostatic, steric and hydrophobic contributions, and (b) the ‘steric’ energetic model. To simplify the latter calculations, only two possible energies were allowed: the interaction energy, E_int_, was set to +∞ if any of the protein's atoms came within 4.5Å of any of the cytoplasm atoms, and was set to zero if not; this binary scoring method is effectively identical to that used in most examinations of excluded-volume (crowding) effects. Due to the very significant computational expense associated with the particle-insertion calculations, they were applied only to the final ‘snapshot’ of the three independent BD simulations performed with the ‘full’ and ‘steric’ models. Error bars for all reported free energy changes were therefore calculated as the standard deviation of the computed values obtained from the three different system ‘snapshots’. The total number of unfolded and folded-state trial positions that were accepted and rejected for each protein, for each of the three ‘full’ model cytoplasm ‘snapshots’ are listed in Table S3.

### Cytoplasm effects on protein association equilibria

A very similar protocol was used to calculate the effects of the cytoplasm on a variety of protein association reactions. Calculations on each assembled protein complex were performed exactly as described above. Calculations on each disassembled complex – e.g. two separated protein monomers in the case of a dimerization reaction – were carried out by performing insertions of all components *simultaneously*; importantly, each randomized placement was first screened to ensure that there were no steric clashes between any of the inserted components *before* their interactions with the cytoplasm were evaluated. As might be expected, the requirement of simultaneously placing multiple molecules into the cytoplasm meant that in some cases very large numbers of trial positions were required in order to obtain reasonably converged results. Owing to the significant computational expense, therefore, calculations were only performed on snapshots taken from BD simulations performed with the ‘full’ energy model. In addition, since the Boltzmann-weighting of the sampled interaction energies can contribute significant noise in cases where the number of accepted placements are comparatively low, the cytoplasm-interaction energy distributions were first smoothed by fitting to sums of three Gaussians using SigmaPlot [98] (see Fig. S9 for a typical fit). The total numbers of accepted and attempted insertions for the various association reactions studied are listed in Table S4.

Dimerization equilibria were investigated by performing separate particle-insertion calculations on the dimeric forms and the monomeric forms; for such calculations it was assumed that no structural change (e.g. unfolding) occurs when the two monomers are separated. The trimerization equilibrium of ParM was investigated in analogous fashion, by performing calculations on a trimer extracted from the ParM filament model (pdbcode: 2QU4 [106]). The aggregation of a poly-Q-inserted RNaseA to form an amyloid fiber was studied using the theoretical model developed by Eisenberg and co-workers (pdbcode: 2APU; [38]). The model deposited in the PDB contains 56 aggregated monomeric units; the largest aggregate for which we could obtain reasonably precise free energy estimates however contained eight monomeric units (Fig. 4F). Since formation of the amyloid structure involves a significant change in conformation, the use of monomeric structures extracted without modification from the aggregate model would be inappropriate. Instead, the structure of the monomeric poly-Q-inserted RNaseA was taken from the crystal structure reported by the Eisenberg group (pdbcode: 2APQ [38]). In order to ensure sequence-consistency with the amyloid model, a A131H mutation was made with SCWRL3 [104]. In addition, since the monomeric structure has no resolved coordinates for the inserted GQQQQQQQQQQGNP stretch this region was model-built using the loop-building program Loopy [107]. The second aggregate structure studied was a theoretical model of SH3 domain aggregation proposed by the Shakhnovich group [39] and kindly made available to the authors by Dr. Feng Ding (UNC; personal communication). This structure contains only C_α_ atoms so complete backbone coordinates were first constructed using the SABBAC webserver [108] (http://bioserv.rpbs.jussieu.fr/cgi-bin/SABBAC) before sidechain positions were constructed using SCWRL3. Owing to the structure's origins being a C_α_-only model we were unable to add sidechains in such a way that the assembled aggregate model was free of internal steric clashes; this, however, does not significantly affect our ability to estimate the model's interaction with the cytoplasm environment. As with the RNaseA amyloid model, it would be inappropriate to assume that the conformations of unaggregated monomeric units are identical to those found in the amyloid model; instead therefore the conformation of the monomeric SH3 domain was taken from the crystal structure (pdbcode: 1NLO [109]).

Two movies, each showing 1.8µs of simulation, are provided as separate Quicktime .mov files. Video S1 shows a BD simulation performed with the ‘full’ energy model; Video S2 shows a BD simulation performed with the ‘steric’ energy model. File size restrictions at the PLoS website have limited the size and resolution of the uploaded movies to be used for review. Higher resolution movies are available to readers at the authors' website: http://dadiddly.biochem.uiowa.edu/Elcock_Lab/Movies.html.

## Supporting Information

Figure S1 Views of the three independent system setups before and after 15µs of BD simulation with the ‘full’ energy model. 50S and 30S ribosomal subunits can be identified by the green/yellow of their RNA and the blue and red (respectively) of their proteins. This figure was prepared with VMD [110].
(3.10 MB TIF)

Figure S2 Total system energy and its electrostatic and hydrophobic components, plotted versus simulation time; the vertical dashed line indicates the beginning of the production simulation.
(0.13 MB TIF)

Figure S3 Histogram of cytoplasm-interaction energies, E_int_, obtained for all non-clashing insertions of the aggregated and non-aggregated states of the SH3 domain.
(0.11 MB TIF)

Figure S4 Time constant for the exponential describing the descent to the minimal value of the anomality exponent, α, plotted for all molecule types versus molecular weight.
(0.09 MB TIF)

Figure S5 Time constant for the exponential describing the return to normal rotational diffusion plotted for all molecule types versus molecular weight; note that for the ‘steric’ model rotational diffusion is essentially normal at almost all observation intervals examined.
(0.09 MB TIF)

Figure S6 Plot showing the quality of fit of a two-exponential decay function to the autocorrelation function describing rotational motion for the three most abundant proteins in the model. Symbols indicate the simulation data; lines indicate the two-exponential fit.
(0.10 MB TIF)

Figure S7 Ratio of the short-time and long-time rotational diffusion coefficients to the infinite-dilution value plotted for the ‘full’ model for all molecule types versus molecular weight.
(0.09 MB TIF)

Figure S8 Plot showing the quality of fit of a two-exponential decay function to the function describing the loss of neighbors for five selected molecule types. Symbols indicate the simulation data; lines indicate the two-exponential fit
(0.10 MB TIF)

Figure S9 Plot showing the quality of fit of a 3-Gaussian distribution to the cytoplasm-interaction energy distributions obtained for non-clashing insertions of the IcdA protein in dimeric and monomeric states; note that the y-axis is on a logarithmic scale.
(0.11 MB TIF)

Table S1 Ordered list of all those proteins identified and quantified in Table 4 of Link et al. [12] under minimal medium conditions and for which the cellular location is either clearly cytoplasmic or undetermined. ‘N-abd’ is the cellular abundance of each chain of the protein determined by Link et al. ‘MW’ is the molecular weight of each chain of the protein as estimated from the amino acid sequence in the Ecocyc database [73]. Asterisks in the ‘Mod.’ column identify those proteins present in our cytoplasm model; note that the low-abundant proteins SucC and RplC are included in the model because they are components of more abundant protein complexes.
(0.25 MB RTF)

Table S2 Alphabetically-ordered list of the macromolecules present in our cytoplasm model showing the pdbcode of their originating structures, the infinite-dilution translational and rotational diffusion coefficients [88], and the sequence coverage of each model.
(1.25 MB PDF)

Table S3 Details of the particle-insertion calculations of the folding equilibria of 8 different proteins, listed in order of increasing protein chain length. Results are shown only for insertions into ‘snapshots’ (A, B, C) taken from BD simulations performed with the ‘full’ energy model. The total numbers of attempted insertions for the folded and unfolded states (for each ‘snapshot’) are 25 million and 250 million respectively. ΔGWidom and ΔΔG are insertion free energies obtained using the ‘steric’ energy model: these numbers can be obtained directly from knowledge of the number of attempted and successful insertions listed in this table.
(0.10 MB RTF)

Table S4 Details of the particle-insertion calculations of the association equilibria of 14 different proteins. ‘Process’ refers to the stoichiometry of the association process examined: 1→2 denotes that the equilibrium is between two monomers and one dimer, 4→8 denotes that the equilibrium is between two tetramers and one octamer etc. As in Table S3, ΔΔG is the insertion free energy difference obtained using the ‘steric’ energy model: this number can be obtained directly from knowledge of the number of attempted and successful insertions listed in this table.
(0.12 MB RTF)

Video S1 Cytoplasm Full Energy Model. 1.8 microseconds of simulation carried out with the ‘full’ energy model.
(9.97 MB MOV)

Video S2 Cytoplasm Steric Energy Model. 1.8 microseconds of simulation carried out with the ‘steric’ energy model.
(9.96 MB MOV)
