# Supplementary figures and images for: Diffusion, Crowding & Protein Stability in a Dynamic Molecular Model of the Bacterial Cytoplasm
Source: PLoS Comput Biol. 2010 Mar 5;6(3):e1000694. doi: 10.1371/journal.pcbi.1000694 (PMC2832674; doi:10.1371/journal.pcbi.1000694)

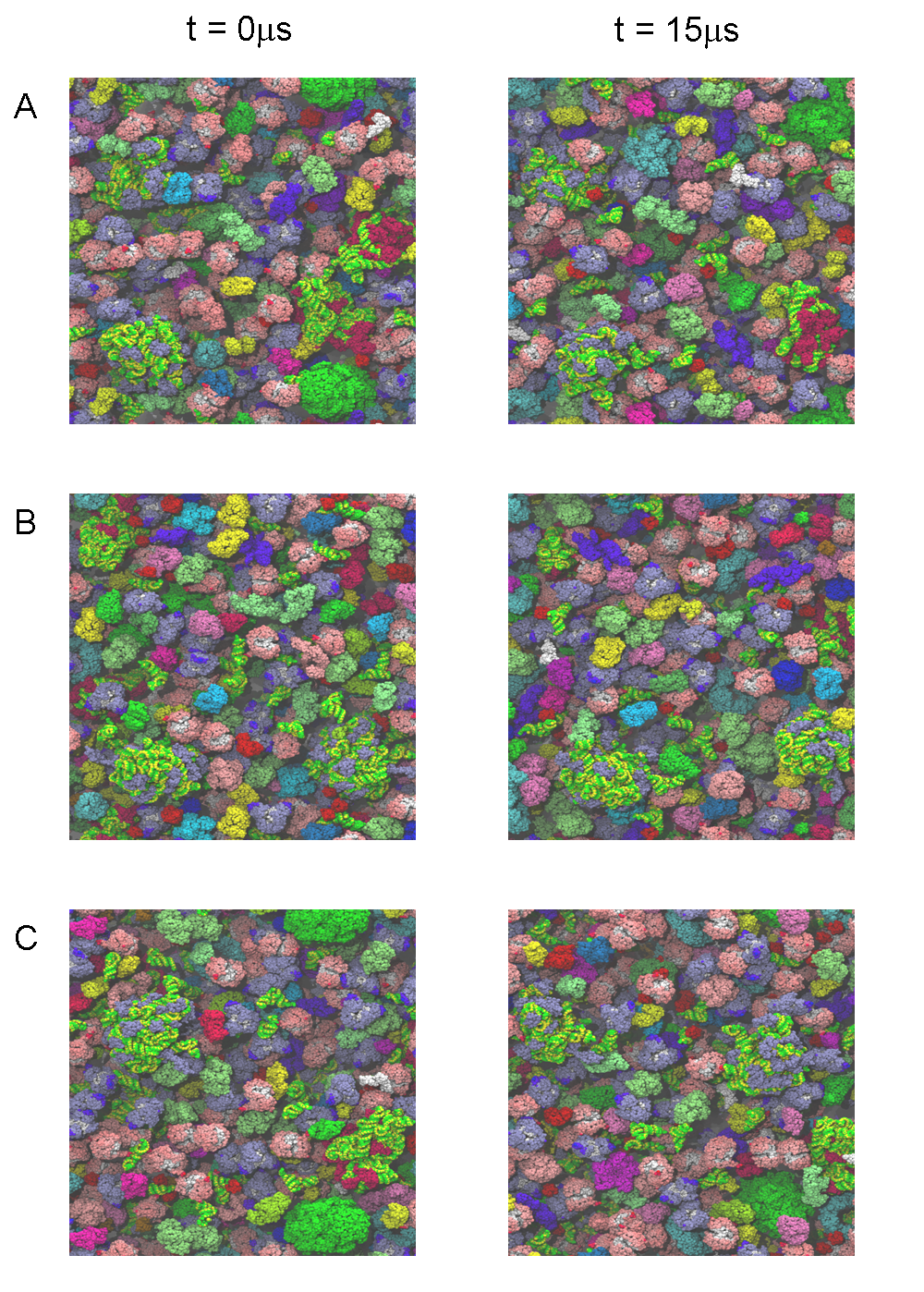

Supplement: Figure S1 — Views of the three independent system setups before and after 15µs of BD simulation with the ‘full’ energy model. 50S and 30S ribosomal subunits can be identified by the green/yellow of their RNA and the blue and red (respectively) of their proteins. This figure was prepared with VMD [110]. (3.10 MB TIF) [file pcbi.1000694.s001.tif]

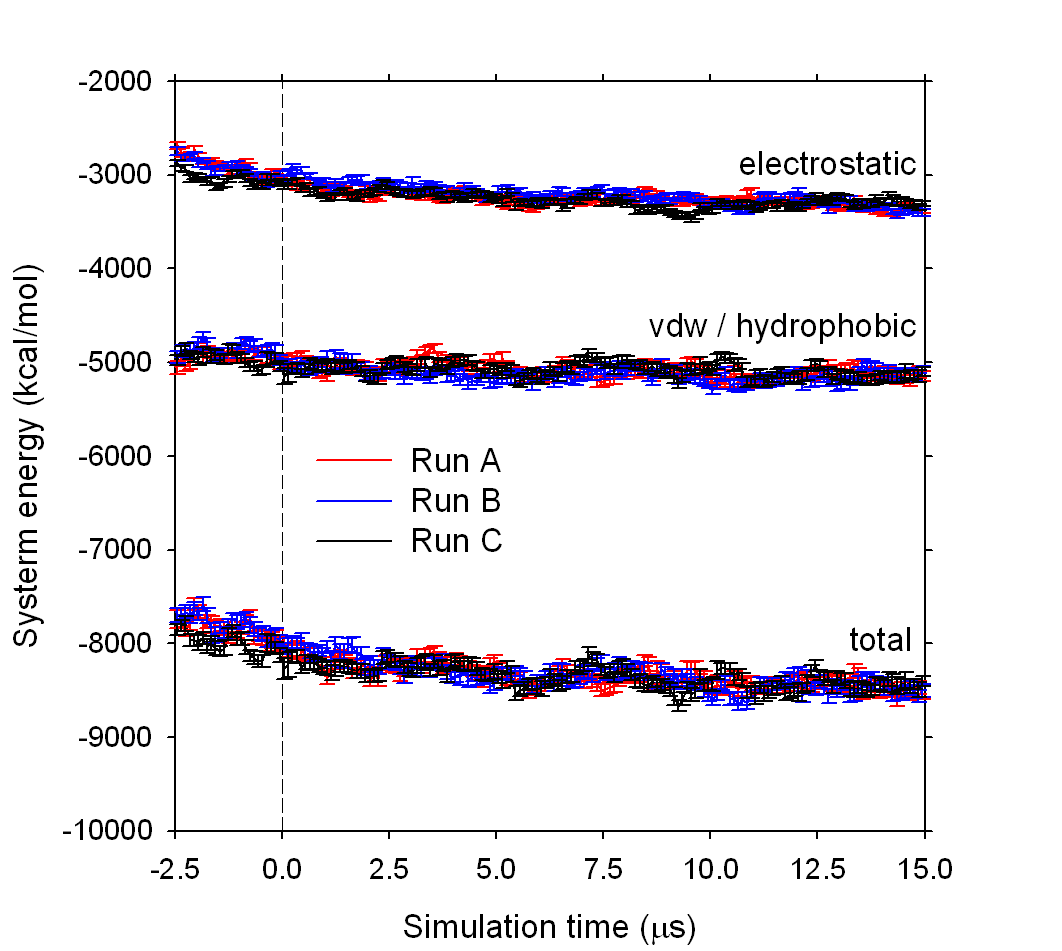

Supplement: Figure S2 — Total system energy and its electrostatic and hydrophobic components, plotted versus simulation time; the vertical dashed line indicates the beginning of the production simulation. (0.13 MB TIF) [file pcbi.1000694.s002.tif]

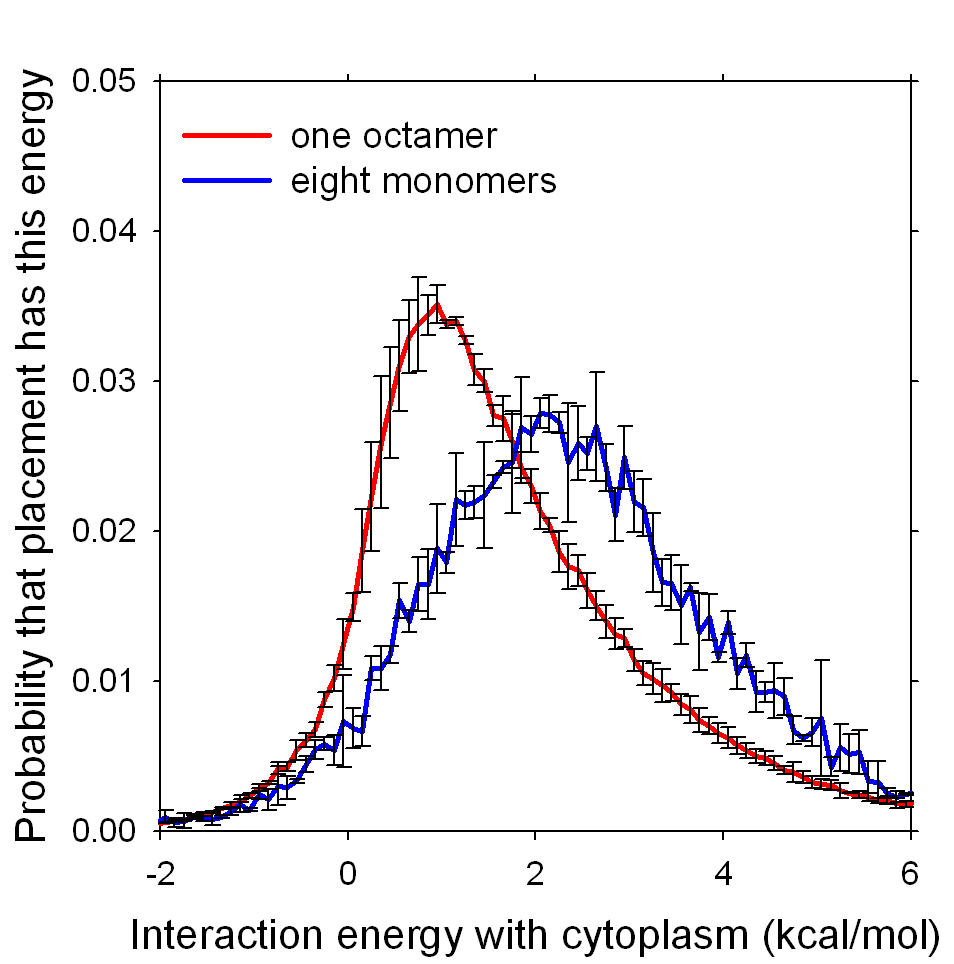

Supplement: Figure S3 — Histogram of cytoplasm-interaction energies, Eint, obtained for all non-clashing insertions of the aggregated and non-aggregated states of the SH3 domain. (0.11 MB TIF) [file pcbi.1000694.s003.tif]

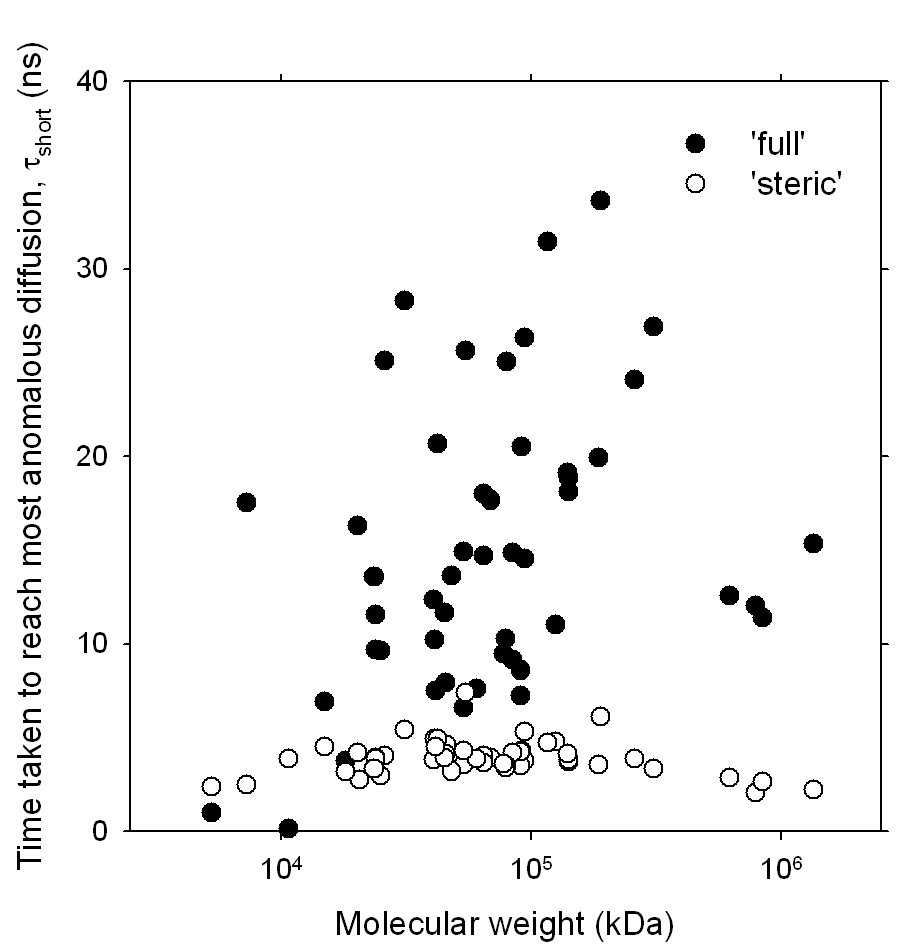

Supplement: Figure S4 — Time constant for the exponential describing the descent to the minimal value of the anomality exponent, α, plotted for all molecule types versus molecular weight. (0.09 MB TIF) [file pcbi.1000694.s004.tif]

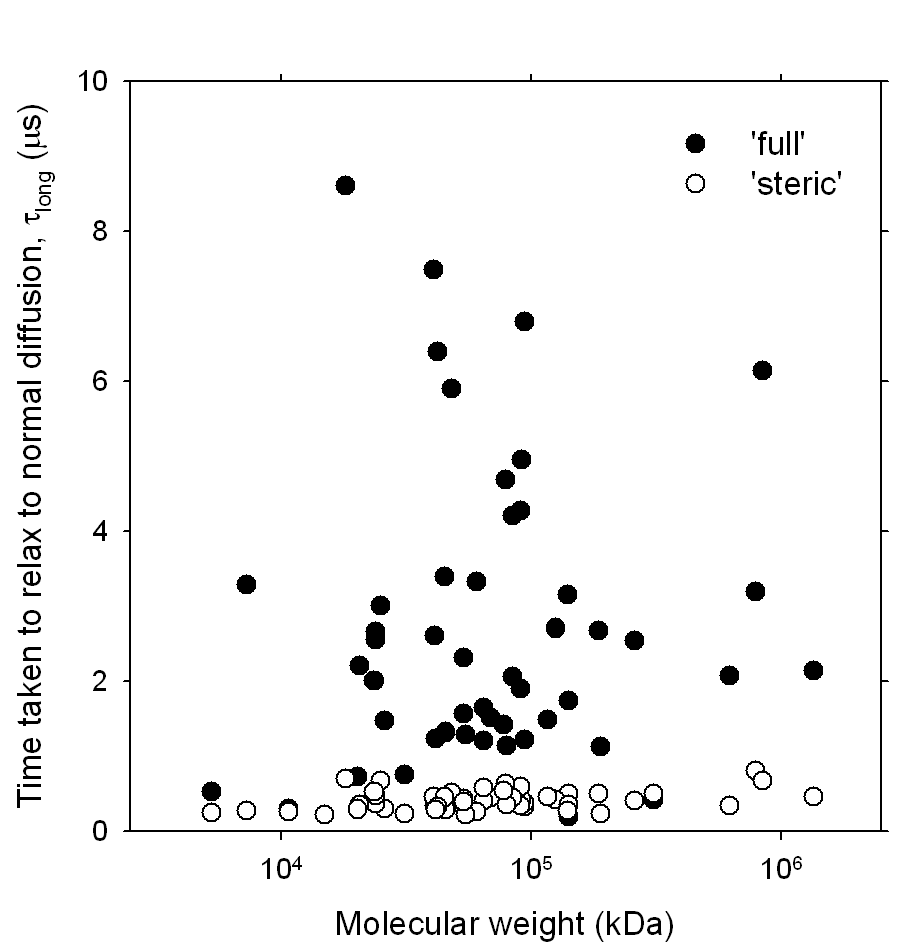

Supplement: Figure S5 — Time constant for the exponential describing the return to normal rotational diffusion plotted for all molecule types versus molecular weight; note that for the ‘steric’ model rotational diffusion is essentially normal at almost all observation intervals examined. (0.09 MB TIF) [file pcbi.1000694.s005.tif]

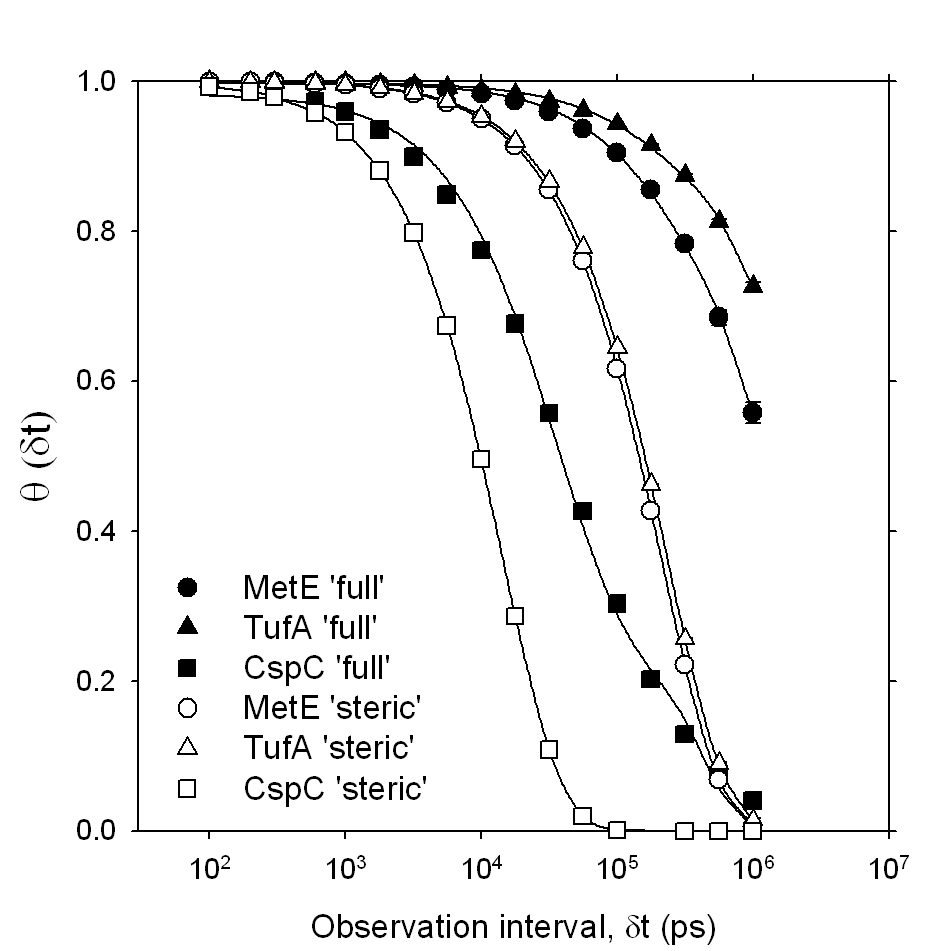

Supplement: Figure S6 — Plot showing the quality of fit of a two-exponential decay function to the autocorrelation function describing rotational motion for the three most abundant proteins in the model. Symbols indicate the simulation data; lines indicate the two-exponential fit. (0.10 MB TIF) [file pcbi.1000694.s006.tif]

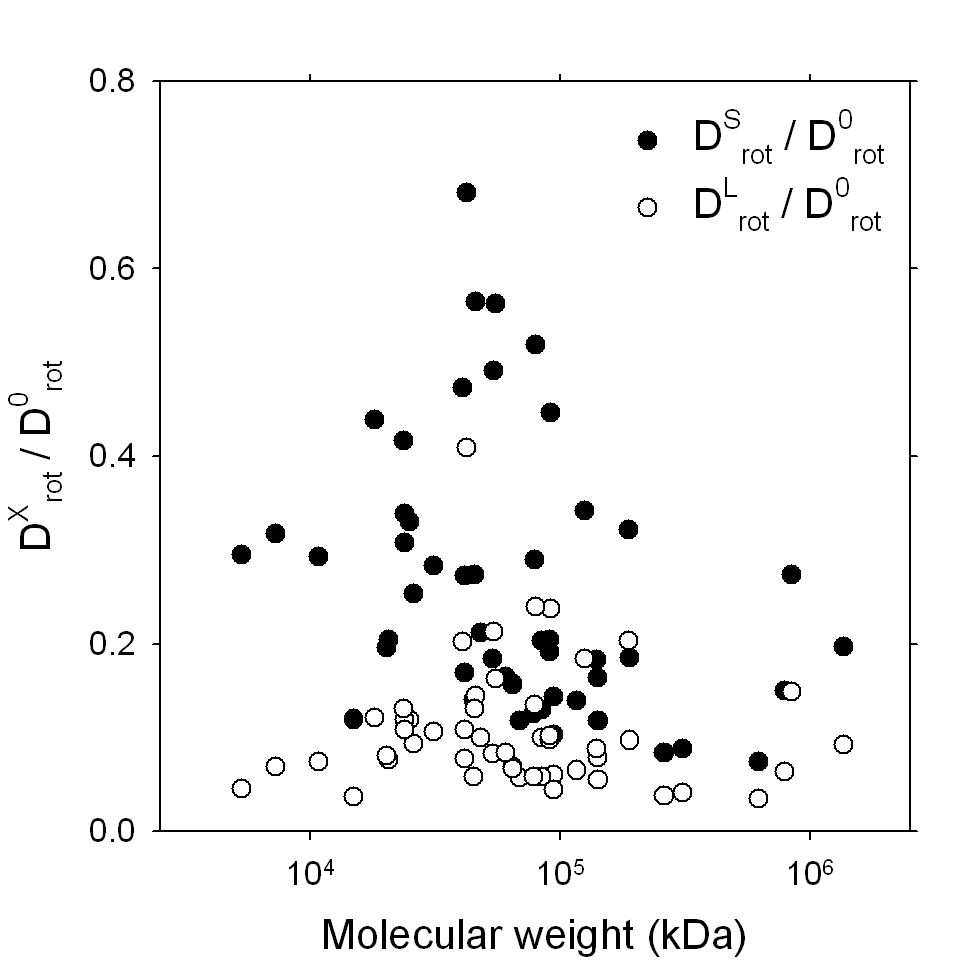

Supplement: Figure S7 — Ratio of the short-time and long-time rotational diffusion coefficients to the infinite-dilution value plotted for the ‘full’ model for all molecule types versus molecular weight. (0.09 MB TIF) [file pcbi.1000694.s007.tif]

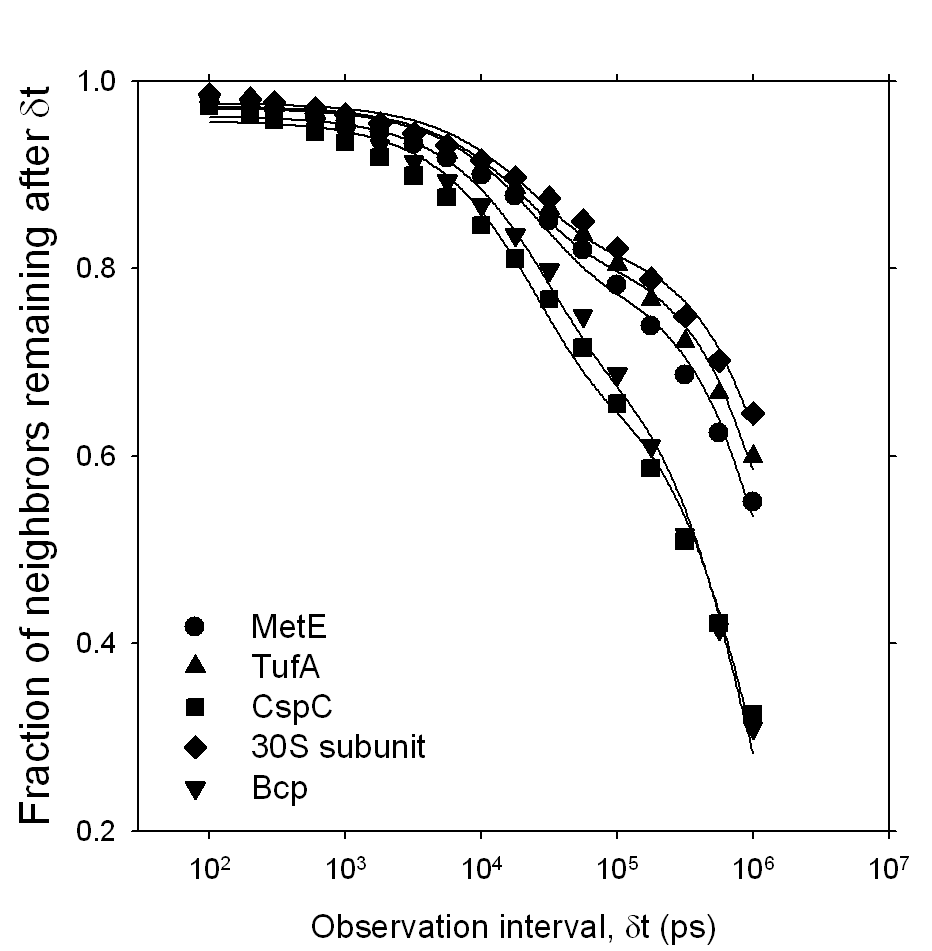

Supplement: Figure S8 — Plot showing the quality of fit of a two-exponential decay function to the function describing the loss of neighbors for five selected molecule types. Symbols indicate the simulation data; lines indicate the two-exponential fit (0.10 MB TIF) [file pcbi.1000694.s008.tif]

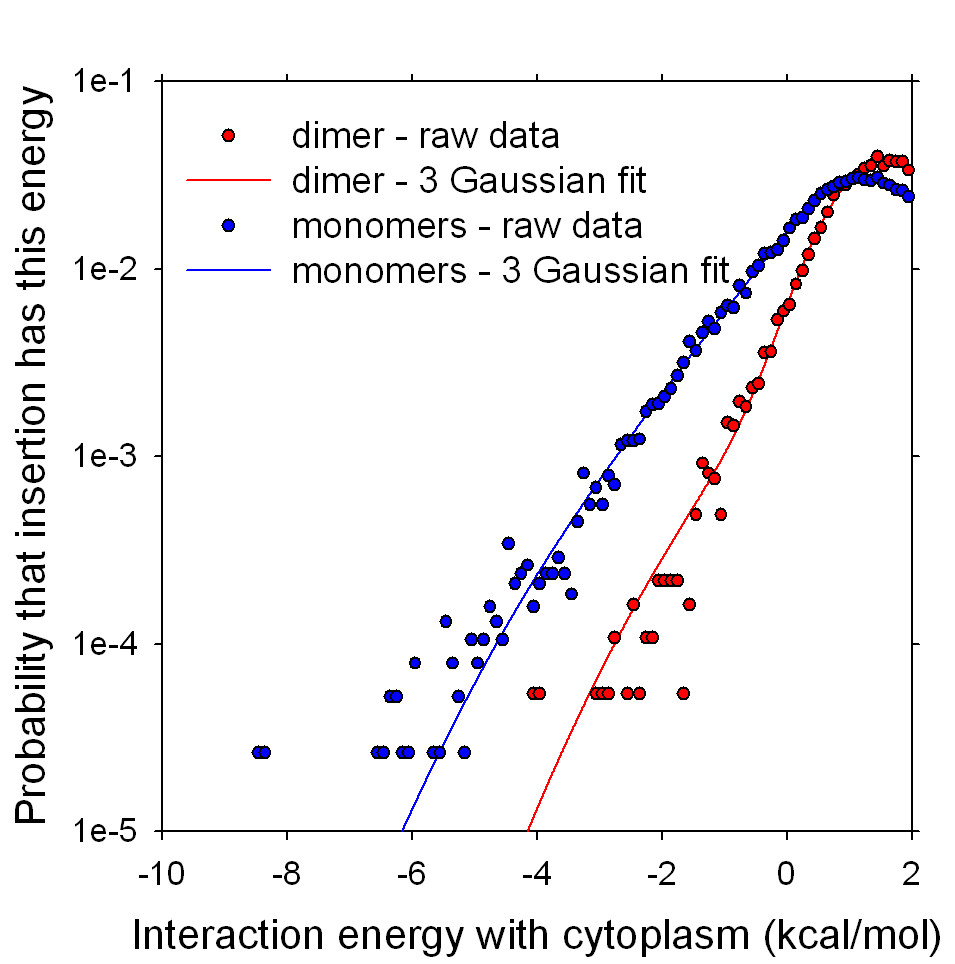

Supplement: Figure S9 — Plot showing the quality of fit of a 3-Gaussian distribution to the cytoplasm-interaction energy distributions obtained for non-clashing insertions of the IcdA protein in dimeric and monomeric states; note that the y-axis is on a logarithmic scale. (0.11 MB TIF) [file pcbi.1000694.s009.tif]
