# Supplementary material for: Diffusion, Crowding & Protein Stability in a Dynamic Molecular Model of the Bacterial Cytoplasm
Source: PLoS Comput Biol. 2010 Mar 5;6(3):e1000694. doi: 10.1371/journal.pcbi.1000694 (PMC2832674; doi:10.1371/journal.pcbi.1000694)
Supplement: Table S1 — Ordered list of all those proteins identified and quantified in Table 4 of Link et al. [12] under minimal medium conditions and for which the cellular location is either clearly cytoplasmic or undetermined. ‘N-abd’ is the cellular abundance of each chain of the protein determined by Link et al. ‘MW’ is the molecular weight of each chain of the protein as estimated from the amino acid sequence in the Ecocyc database [73]. Asterisks in the ‘Mod.’ column identify those proteins present in our cytoplasm model; note that the low-abundant proteins SucC and RplC are included in the model because they are components of more abundant protein complexes. (0.25 MB RTF) [file pcbi.1000694.s010.rtf]

Protein	N-abd	MW	Mod.	Protein	N-abd	MW	Mod.	Protein	N-abd	MW	Mod.	
TufA	41400	43.3	*	PanB	1080	28.2	*	ArgG	380	49.9		
MetE	24420	84.7	*	Tig	1070	48.2	*	SerA	380	44.2		
IcdA	9900	45.8	*	Pnp	1000	26.0	*	TrpB	360	42.9		
AhpC	8590	20.8	*	MopB	980	10.4	*	IlvI	360	63.0		
CspC	8300	7.4	*	GpmA	960	28.6	*	PtsI	360	63.6		
RpsB	8100	26.7	*	PurA	940	47.3	*	AroK	340	19.5		
Ppa	5980	19.7	*	SucD	900	29.8	*	PyrC	340	38.8		
RplD	4960	22.1	*	Asd	900	41.1	*	NusA	340	54.9		
GapA	4460	35.5	*	HupB	860	9.2	*	YifE	340	13.1		
Eno	4020	45.7	*	RpoA	850	36.5	*	YihK	340	67.4		
GlyA	3480	45.3	*	DapA	840	31.3	*	SucC	340	41.4	*	
RplI	3400	15.8	*	Frr	840	20.6	*	FtsZ	320	40.3		
CysK	3060	34.5	*	Hns	820	15.5	*	Rho	300	47.0		
Pgk	2990	41.1	*	ProS	800	63.7		AldA	280	52.3		
SodA	2980	23.1	*	GltD	800	52.0	*	TrpA	280	28.7		
Mdh	2900	32.3	*	PpiB	780	18.2	*	LeuC	280	49.9		
Tsf	2670	30.4	*	RpiA	740	22.9	*	NfnB	260	23.9		
RpsF	2620	15.2	*	CysI	640	64.0		DksA	260	17.5		
Upp	2580	22.5	*	PurH	640	57.3		YchF	260	39.7		
RplM	2580	16.0	*	HtpG	640	71.4		YebL	240	33.8		
MopA	2540	57.3	*	CarA	620	41.4		HisD	220	46.1		
FusA	2520	77.6	*	AroG	600	38.0		ArgD	200	43.8		
SerC	2500	39.8	*	GuaB	600	52.0		ArcA	200	27.3		
PyrB	2080	34.4	*	YigW	600	29.0		PurM	180	36.9		
IlvC	2060	54.1	*	ThrC	560	47.1		AccC	180	49.3		
SodB	2020	21.3	*	YeaD	560	32.7		AccA	180	35.2		
PurC	1680	27.0	*	LpdA	540	50.7		YhfO	180	31.2		
UspA	1660	16.1	*	GalU	520	32.9		RplC	180	22.2	*	
Adk	1640	23.6	*	GlnS	520	63.5		FumA	160	60.3		
Efp	1600	20.6	*	PykF	500	50.7		LeuA	160	57.3		
Fba	1480	39.1	*	FabD	500	32.4		PrsA	160	34.2		
GlnA	1460	51.9	*	PyrG	480	60.4		GuaC	160	37.4		
HupA	1380	9.5	*	SspA	480	24.3		YiaE	160	35.4		
RpsA	1360	61.2	*	AspC	460	43.6		YbiS	160	33.3		
YjgF	1320	13.6		FabI	440	27.9		ArgI	140	36.9		
YdfG	1300	27.2		YgaG	440	19.4		GdhA	140	48.6		
DnaK	1300	69.1	*	SucB	420	44.0		YjjK	140	62.4		
YacI	1260	93.5		DapD	420	29.9		ValS	120	108.2		
RpsP	1220	9.2	*	FolE	420	24.8		NadE	100	30.6		
PyrI	1200	17.1	*	LeuB	400	39.5		AsnS	80	52.6		
AceE	1140	99.7		GlyS	400	76.8						
TpiA	1120	27.0	*	TalB	380	35.2						

Table S1
