# Supplementary material for: Diffusion, Crowding & Protein Stability in a Dynamic Molecular Model of the Bacterial Cytoplasm
Source: PLoS Comput Biol. 2010 Mar 5;6(3):e1000694. doi: 10.1371/journal.pcbi.1000694 (PMC2832674; doi:10.1371/journal.pcbi.1000694)
Supplement: Table S2 — Alphabetically-ordered list of the macromolecules present in our cytoplasm model showing the pdbcode of their originating structures, the infinite-dilution translational and rotational diffusion coefficients [88], and the sequence coverage of each model. (1.25 MB PDF) [file pcbi.1000694.s011.pdf]

Image	Protein Name	Subunit Composition		PDB	Mw (Da)	Total Mw (Da)	# in Sim.	Dtrans ($Å^2$/ns)	Drot (/ns)	% Identity
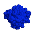	Adk	1	Adk	1AKE	23,585	23,585	14	9.04	0.01144	100%
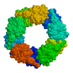	AhpC	10	AhpC	1YEP	18,720	187,200	7	4.03	0.00096	90%
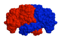	Asd	2	Asd	1BRM	40,016	80,031	4	5.90	0.00313	100%
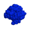	Bcp	1	Bcp	1XXU	10,757	10,757	8	11.26	0.02202	62%
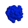	CspC	1	CspC	1MJC	7,271	7,271	72	13.23	0.03589	100%
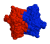	CysK	2	CysK	1FCJ	32,257	64,515	13	6.36	0.00391	94%
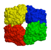	DapA	4	DapA	1DHP	31,268	125,073	2	5.24	0.00222	100%
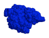	DnaK	1	DnaK	1DKG	41,319	41,319	11	7.27	0.00579	60%
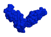	Efp	1	Efp	1YBY	20,260	20,260	14	8.11	0.00772	98%
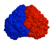	Eno	2	Eno	1E9I	45,450	90,900	18	5.91	0.00321	100%
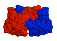	Fba	2	Fba	1GYN	39,014	78,028	6	5.92	0.00314	100%
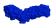	Frr	1	Frr	1EK8	20,637	20,637	7	8.00	0.00829	100%
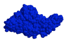	FusA	1	FusA	1KTV	68,825	68,825	22	5.64	0.00273	89%
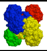	GapA	4	GapA	1S7C	35,399	141,598	10	4.98	0.00192	100%
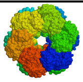	GlnA	12	GlnA	2GLS	51,770	621,238	1	2.99	0.00042	100%
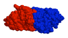	GltD	2	GltD	1H7X	46,977	93,954	3	5.20	0.00211	91%
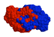	GlyA	2	GlyA	1DFO	45,314	90,629	15	5.98	0.00329	100%
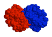	GpmA	2	GpmA	1E59	27,485	54,969	4	6.54	0.00427	96%
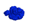	Hns	1	Hns	1HNR	5,301	5,301	7	13.99	0.04131	100%
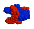	Hup	1	HupA	1MUL	7,955	14,956	12	10.12	0.01585	84%
		1	HupB	1MUL	7,002					76%
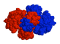	IcdA	2	IcdA	1P8F	45,754	91,509	43	5.65	0.00275	100%
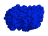	IlvC	1	IlvC	1YRL	53,736	53,736	18	6.90	0.00503	99%
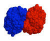	Mdh	2	Mdh	2CMD	32,336	64,671	13	6.42	0.00407	100%
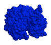	MetE	1	MetE	1U22	84,323	84,323	213	5.70	0.00282	100%
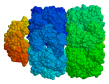	Mop	14	MopA	1XCK	55,155	844,879	2	2.54	0.00025	96%
		7	MopB	1PCQ	10,386					100%
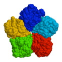	PanB	5	PanB	1M3U	27,977	139,883	2	4.73	0.00158	99%
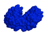	Pgk	1	Pgk	1PHP	40,788	40,788	26	7.32	0.00606	99%

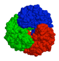	Pnp	3	Pnp	1E3P	63,171	189,513	3	4.37	0.00130	79%
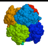	Ppa	6	Ppa	2EIP	19,329	115,974	9	5.39	0.00245	98%
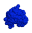	PpiB	1	PpiB	1LOP	18,153	18,153	7	10.14	0.01624	100%
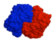	PurA	2	PurA	1ADE	47,211	94,422	4	5.73	0.00290	100%
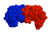	PurC	2	PurC	1KUT	20,750	41,500	7	6.78	0.00468	77%
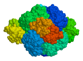	Pyr	6	PyrB	1ZA1	34,294	308,484	3	3.65	0.00072	100%
		6	PyrI	2A0F	17,120					100%
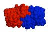	RpiA	2	RpiA	1KS2	22,728	45,456	3	7.03	0.00532	100%
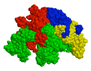	Rpo	2	RpoA	2CW0	25,649	260,123	4	3.58	0.00069	71%
		1	RpoB	2BE5	109,580					73%
		1	RpoC	1HQM	99,245					64%
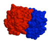	SerC	2	SerC	1BJO	39,579	79,158	11	5.99	0.00327	99%
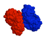	SodA	2	SodA	1D5N	22,965	45,929	13	7.19	0.00572	100%
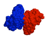	SodB	2	SodB	1ISC	21,133	42,267	9	7.32	0.00601	99%
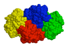	Suc	2	SucC	1JLL	41,390	141,814	4	4.82	0.00172	100%
		2	SucD	1JLL	29,517					99%
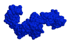	Tig	1	Tig	1W26	47,988	47,988	9	5.66	0.00269	100%
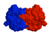	TpiA	2	TpiA	1TRE	26,970	53,941	5	6.78	0.00480	100%
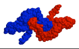	Tsf	2	Tsf	1EFU	30,290	60,580	12	5.46	0.00242	100%
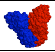	TufA	2	TufA	1DG1	42,144	84,288	181	5.62	0.00269	98%
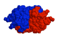	Upp	2	Upp	1I5E	22,532	45,064	11	6.78	0.00473	100%
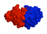	UspA	2	UspA	1JMV	15,547	31,094	7	7.99	0.00784	97%
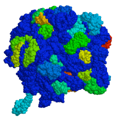	50S	1	5S RNA	2AW4	38,790	1,355,089	10	2.19	0.00016	96%
		1	23S RNA	2AW4	941,614					97%
		29	L2-6, L9,L11, L13-25, L27, L29-36 Proteins	2AW4	374,685					99%

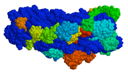	30S	1	16S RNA	2AVY	499,691	787,666	10	2.56	0.00025	99%
		20	S2-21 Proteins	2AVY	287,974					91%
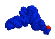	tRNA-CYS	1	tRNA-CYS	1B23	23,914	23,914	37	7.78	0.00671	84%
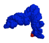	tRNA-GLN	1	tRNA-GLN	1EUY	23,817	23,817	37	7.88	0.00676	60%
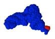	tRNA-PHE	1	tRNA-PHE	1OB2	25,007	25,007	37	7.71	0.00655	99%
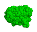	WT-GFP	1	GFP	1GFL	25,900	25,900	8	8.86	0.01072	97%

Table S2
