# Supplementary material for: Diffusion, Crowding & Protein Stability in a Dynamic Molecular Model of the Bacterial Cytoplasm
Source: PLoS Comput Biol. 2010 Mar 5;6(3):e1000694. doi: 10.1371/journal.pcbi.1000694 (PMC2832674; doi:10.1371/journal.pcbi.1000694)
Supplement: Table S3 — Details of the particle-insertion calculations of the folding equilibria of 8 different proteins, listed in order of increasing protein chain length. Results are shown only for insertions into ‘snapshots’ (A, B, C) taken from BD simulations performed with the ‘full’ energy model. The total numbers of attempted insertions for the folded and unfolded states (for each ‘snapshot’) are 25 million and 250 million respectively. ΔGWidom and ΔΔG are insertion free energies obtained using the ‘steric’ energy model: these numbers can be obtained directly from knowledge of the number of attempted and successful insertions listed in this table. (0.10 MB RTF) [file pcbi.1000694.s012.rtf]

Protein	#res	Rgyr Folded (Å)	# Success Folded (millions)	ΔGWidom Folded ('steric')	# Success Unfolded (millions)	ΔGWidom Unfolded ('steric')	ΔΔG ('steric')	
			A	B	C		A	B	C			
CspC	68	11.1	4.66	4.69	4.61	1.00	13.27	13.61	12.91	1.74	0.74	
6-85	80	12.3	4.02	4.06	3.97	1.08	7.68	7.98	7.45	2.06	0.98	
Bcp	97	13.3	3.18	3.22	3.12	1.22	4.96	5.23	4.80	2.32	1.10	
Crb	136	14.9	2.47	2.41	2.37	1.38	1.74	1.58	1.52	2.99	1.61	
PpiB	164	14.8	2.27	2.31	2.21	1.42	0.72	0.82	0.69	3.45	2.02	
Efp	185	23.2	0.94	0.98	0.91	1.94	0.42	0.50	0.41	3.76	1.81	
Adk	214	16.7	1.50	1.56	1.46	1.66	0.16	0.20	0.15	4.32	2.66	
GFP	230	17.1	1.41	1.47	1.37	1.70	0.14	0.17	0.14	4.41	2.71	


Table S3
