# Supplementary material for: Diffusion, Crowding & Protein Stability in a Dynamic Molecular Model of the Bacterial Cytoplasm
Source: PLoS Comput Biol. 2010 Mar 5;6(3):e1000694. doi: 10.1371/journal.pcbi.1000694 (PMC2832674; doi:10.1371/journal.pcbi.1000694)
Supplement: Table S4 — Details of the particle-insertion calculations of the association equilibria of 14 different proteins. ‘Process’ refers to the stoichiometry of the association process examined: 1→2 denotes that the equilibrium is between two monomers and one dimer, 4→8 denotes that the equilibrium is between two tetramers and one octamer etc. As in Table S3, ΔΔG is the insertion free energy difference obtained using the ‘steric’ energy model: this number can be obtained directly from knowledge of the number of attempted and successful insertions listed in this table. (0.12 MB RTF) [file pcbi.1000694.s013.rtf]

Protein	#res	process	# Success  Oligomer (thousands)	# Attempts  Oligomers (millions)  	# Success  Monomers  (thousands)	# Attempts  Monomers (millions)  	ΔΔG 'steric'	
			A	B	C		A	B	C			
PurC	182	12	114	130	113	10	79	86	76	25	-0.77	
SodB	192	12	183	204	179	10	125	132	118	25	-0.79	
Upp	208	12	118	119	118	10	40	40	41	25	-1.18	
RpiA	218	12	156	175	155	10	97	105	92	25	-0.84	
SodA	205	12	170	193	168	10	111	119	105	25	-0.82	
TufA	385	12	32	45	35	20	58	73	56	250	-1.19	
GlyA	417	12	31	41	33	10	60	77	59	250	-1.54	
Eno	430	12	29	39	31	10	103	129	101	250	-1.19	
IcdA	416	12	19	26	21	10	38	51	37	250	-1.51	
GltD	431	12	76	115	87	100	31	42	31	250	-1.13	
PurA	431	12	19	26	21	10	52	67	51	250	-1.35	
ParM	320	13	20	35	25	100	10	14	9	2000	-2.29	
RNaseA	136	14	5	6	5	2	13	14	12	200	-2.24	
RNaseA	136	48	7	12	8	100	7	10	7	1000	-1.42	
SH3	56	18	72	86	73	10	4	4	3	1000	-4.56	


Table S4
